# Wnt/β‐catenin/RAS signaling mediates age‐related renal fibrosis and is associated with mitochondrial dysfunction

**DOI:** 10.1111/acel.13004

**Published:** 2019-07-18

**Authors:** Jinhua Miao, Jiafeng Liu, Jing Niu, Yunfang Zhang, Weiwei Shen, Congwei Luo, Yahong Liu, Chuanjiang Li, Hongyan Li, Peiliang Yang, Youhua Liu, Fan Fan Hou, Lili Zhou

**Affiliations:** ^1^ Division of Nephrology State Key Laboratory of Organ Failure Research, National Clinical Research Center of Kidney Disease, Nanfang Hospital Southern Medical University Guangzhou China; ^2^ Department of Nephrology, Huadu District People’s Hospital Southern Medical University Guangzhou China; ^3^ Department of Hepatobiliary Surgery, Nanfang Hospital Southern Medical University Guangzhou China

**Keywords:** aging, mitochondrial dysfunction, renal fibrosis, Wnt/β‐catenin

## Abstract

Renal fibrosis is the common pathological feature in a variety of chronic kidney diseases. Aging is highly associated with the progression of renal fibrosis. Among several determinants, mitochondrial dysfunction plays an important role in aging. However, the underlying mechanisms of mitochondrial dysfunction in age‐related renal fibrosis are not elucidated. Herein, we found that Wnt/β‐catenin signaling and renin–angiotensin system (RAS) activity were upregulated in aging kidneys. Concomitantly, mitochondrial mass and functions were impaired with aging. Ectopic expression of Klotho, an antagonist of endogenous Wnt/β‐catenin activity, abolished renal fibrosis in d‐galactose (d‐gal)‐induced accelerated aging mouse model and significantly protected renal mitochondrial functions by preserving mass and diminishing the production of reactive oxygen species. In an established aging mouse model, dickkopf 1, a more specific Wnt inhibitor, and the mitochondria‐targeted antioxidant mitoquinone restored mitochondrial mass and attenuated tubular senescence and renal fibrosis. In a human proximal tubular cell line (HKC‐8), ectopic expression of Wnt1 decreased biogenesis and induced dysfunction of mitochondria, and triggered cellular senescence. Moreover, d‐gal triggered the transduction of Wnt/β‐catenin signaling, which further activated angiotensin type 1 receptor (AT1), and then decreased the mitochondrial mass and increased cellular senescence in HKC‐8 cells and primary cultured renal tubular cells. These effects were inhibited by AT1 blocker of losartan. These results suggest inhibition of Wnt/β‐catenin signaling and the RAS could slow the onset of age‐related mitochondrial dysfunction and renal fibrosis. Taken together, our results indicate that Wnt/β‐catenin/RAS signaling mediates age‐related renal fibrosis and is associated with mitochondrial dysfunction.

## 1 INTRODUCTION

Chronic kidney disease (CKD) has a rapidly rising worldwide prevalence (Bruck et al., 2016; Ku, Johansen, & McCulloch, 2018; Yan et al., 2018). Moreover, CKD patients are at particularly high risk of death due to comorbidities such as infection, hypertension, atherosclerosis, heart failure, and stroke (DeBoer et al., 2018; Xu et al., 2016). However, to date, no completely effective therapeutic strategies for CKD have been investigated.

In the etiology of CKD, aging is a particular trigger and sustaining factor (Minutolo, Borrelli, & De Nicola, 2015). Recent studies have shown, compared with youth, the age group of over 70 years old with CKD suffers up to 13‐fold higher morbidity regardless of the country‐specific prevalence (Minutolo et al., 2015). At the age of 50–60 years, human kidneys appear with macrostructural changes in decreased cortical volume, increased surface roughness, and augmented numbers and sizes of cysts (Hommos, Glassock, & Rule, 2017). In aging kidneys, those macrostructural changes correspond to the typical microstructural histological features of glomerulosclerosis and interstitial fibrosis, especially tubular atrophy or senescence (Rule et al., 2010). Of note, senescent tubular cells lose their capacity of self‐repair and produce proinflammatory cytokines and matrix‐synthesizing molecules, which would further accelerate renal fibrosis (Luo et al., 2018). Interestingly, recent studies show that CKD is characterized by many features of aging. These suggest CKD is a clinical presentation of premature aging (Luo et al., 2018; Stenvinkel & Larsson, 2013; Sturmlechner, Durik, Sieben, Baker, & Deursen, 2017). Hence, a better understanding of the mechanisms of age‐related renal fibrosis is important to the discovery of targeted therapeutics for CKD.

Renal tubular cells, which represent the major type of kidney parenchymal cells, execute fundamental functions of reabsorption and secretion in maintaining fluid and electrolyte balance (Wang & Kestenbaum, 2018). These functions impart to tubular cells at high energy consumption. Because the mitochondria serve an essential role in energy production, their normal functions are vital to cellular homeostasis in normal organs (Higgins & Coughlan, 2014; Tang & Dong, 2016). In a variety of acute and chronic kidney diseases, mitochondrial dysfunction could be seen initially and predominantly in tubular cells (Chung et al., 2018; Dare et al., 2015; Kang et al., 2015; Xiao et al., 2017; Yamamoto et al., 2016). It is noted that the morphological changes and loss of function in mitochondria would lead to reactive oxygen species (ROS) production to trigger oxidative stress and inflammation, two major factors in accelerating the progression of renal fibrosis (Feng et al., 2018; Zhou et al., 2012). These findings suggest mitochondrial quality control plays a crucial role in maintaining normal kidney function. However, the underlying mechanisms of mitochondrial dysfunction in renal tubular cells have not been elucidated.

Wnt/β‐catenin signaling, a conserved signaling pathway in organ development, is kept silent in normal adult kidneys (Wang, Zhou, & Liu, 2018). In a variety of CKD models, it is reactivated in damaged kidney cells, predominantly in tubular epithelial cells (He et al., 2009; Zhou, Li, et al., 2015; Zhou, Li, Zhou, Tan, & Liu, 2013; Zhou, Mo, et al., 2015). Importantly, the activation of Wnt/β‐catenin signaling triggers tubular epithelial cell transition to mesenchymal or senescent phenotype, and promotes renal fibrosis (Luo et al., 2018). However, whether the Wnt/β‐catenin promotion of tubular cell senescence is associated with mitochondrial dysfunction remains poorly understood.

In this study, we detected that Wnt/β‐catenin signaling and RAS activity were activated in aging kidneys and senescent tubular epithelial cells in culture. Inhibition of Wnt/β‐catenin and RAS significantly protected normal structures and functions of mitochondria, and mitigated age‐related renal fibrosis. Our results suggest that Wnt/β‐catenin/RAS signaling plays a crucial role in mediating age‐related renal fibrosis and is associated with mitochondrial dysfunction.

## 2 RESULTS

### 2.1 Wnt/β‐catenin signaling and RAS activity are time‐dependently induced in kidneys during aging

To investigate age‐related renal fibrosis, the studies were performed in 7‐, 12‐, and 24‐month‐old mice. First, we tested the expression of Klotho, an anti‐aging protein, which is highly expressed in all major tubular segments in normal adult kidneys (Hu & Moe, 2012; Zhou et al., 2013). As shown in Figure S1a,b, Klotho expression significantly decreased by 12 months of age and further reduced by 24 months of age. Next, we analyzed the tubular injury marker Kim1 (kidney injury molecule‐1), and fibrotic proteins α‐SMA and fibronectin. As shown in Figure S1a,c–e, they were continuously upregulated during the progression of aging. Similar results were demonstrated by analysis of immunohistochemical staining for Klotho and Masson trichrome staining (Figure S1f).

**Figure 1:**
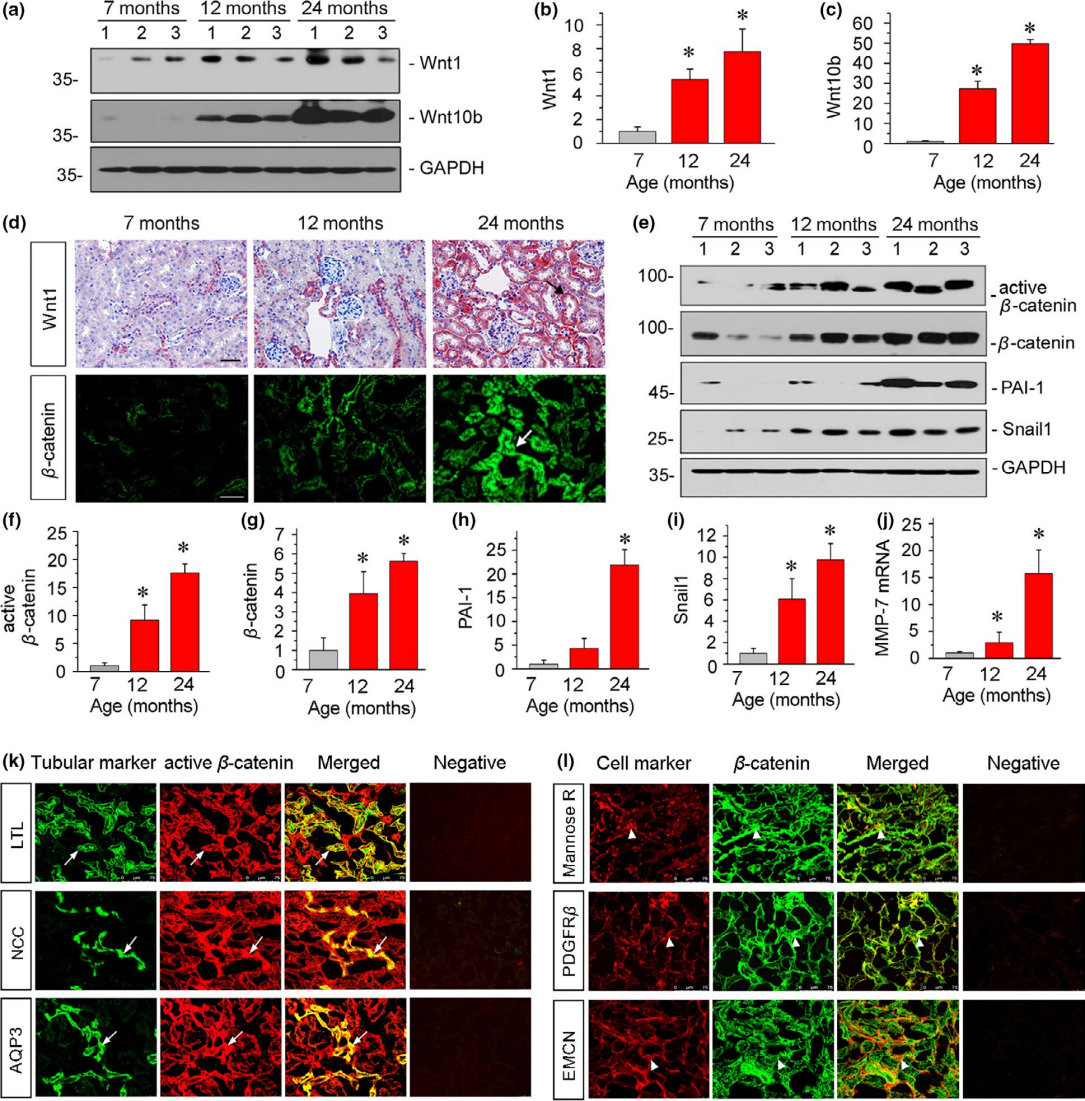
Renal Wnt/β‐catenin signaling is time‐dependently induced in kidneys during aging. (a–c) Western blot analyses show renal expression of Wnt1 and Wnt10b. Graphical representations of (b) Wnt1 and (c) Wnt10b protein expression levels in different groups are presented. *p < .05 versus 7‐month‐old mice (*n* = 5–6). (d) Representative micrographs show renal expression of Wnt1 and β‐catenin. Arrows indicate positive staining. Scale bar, 50 µm. (e–i) Western blot analyses show renal expression of the active form and total β‐catenin, as well as its targets PAI‐1 and Snail1. Graphical representations of (f) active β‐catenin, (g) β‐catenin, (h) PAI‐1, and (i) Snail1 protein expression levels in different groups are presented. *p < .05 versus 7‐month‐old mice (*n* = 5–6). (j) Graphical representation of the relative abundance of MMP‐7 mRNA. *p < .05 versus 7‐month‐old mice (*n* = 5–6). (k) Colocalization of active β‐catenin and various segment‐specific tubular markers in aging kidneys. Frozen kidney sections from 24‐month‐old mice were immunostained for various segment‐specific tubular markers (green) and active β‐catenin (red). Segment‐specific tubular markers were used as follows: proximal tubule, Lotus tetragonolobus lectin (LTL); distal tubule, thiazide‐sensitive NaCl cotransporter (NCC); and collecting duct, aquaporin‐3 (AQP3). Sections incubated with secondary antibodies alone (omitting primary antibody) were used as negative controls (right panel). Arrows indicate positive tubules with colocalization of active β‐catenin and specific tubular makers. Scale bar, 75 μm. (l) Colocalization of β‐catenin and multiple cell type markers in kidneys from 24‐month‐old mice. Frozen kidney sections were co‐stained with various cell type‐specific markers (red) and β‐catenin (green). Cell markers were used as follows: macrophage, mannose R; fibroblasts, PDGFRβ; and endothelial cell, EMCN. Sections incubated with secondary antibodies alone (omitting primary antibody) were used as negative controls (right panel). Arrowheads indicate colocalization of β‐catenin and specific cell makers. Scale bar, 75 μm

We then performed a comprehensive analysis of the mRNA expression of all Wnts. As shown in Figure S2, among 19 Wnts in mammals (Moon, Kohn, De Ferrari, & Kaykas, 2004), 15 Wnts were upregulated in aging kidneys to varying degrees. The protein expression levels of Wnt1 and Wnt10b were also significantly upregulated in aged mice (Figure 1a–c). To confirm the location of Wnt1, we assessed it by immunostaining. As shown in Figure 1d, Wnt1 expression was induced predominantly in renal tubular cells. Similar results were observed when β‐catenin, the downstream effector of Wnt signaling, was assessed (Figure 1d). Next, we analyzed the activation of β‐catenin signaling. As shown in Figure 1e–g, the protein expression levels of the active and total form of β‐catenin were significantly upregulated following the development of aging. We then inspected PAI‐1, Snail1, and MMP‐7, the three important targets that reflect the activity of Wnt/β‐catenin signaling (He et al., 2010, 2012; Simon‐Tillaux & Hertig, 2017). As shown in Figure 1e,h,i, the expression levels of PAI‐1 and Snail1 were significantly induced in aging mice, especially in 24‐month‐old mice. A similar result was observed when MMP‐7 mRNA was assessed by quantitative real‐time PCR (Figure 1j).

To clarify the locations of β‐catenin, we performed the co‐staining in 24‐month‐old mice for active β‐catenin with different segment‐specific tubular cell markers of Lotus tetragonolobus lectin (LTL), a proximal tubular marker, sodium chloride cotransporter (NCC), a marker of distal convoluted tubules, and aquaporin‐3 (AQP3), a marker of collecting duct epithelium. As shown in Figure 1k, active β‐catenin colocalized with different tubular segments. We next carried out double staining for β‐catenin with other cell type markers. In 24‐month‐old mice, renal expression of β‐catenin had colocalization with mannose R, an M2 macrophage marker, platelet‐derived growth factor receptor‐β (PDGFRβ), a fibroblast marker, and EMCN, an endothelial cell marker, respectively (Figure 1l). These data indicate that β‐catenin is widely expressed by multiple cell types in aging kidneys, although it is predominantly located in tubular cells.

**Figure 2:**
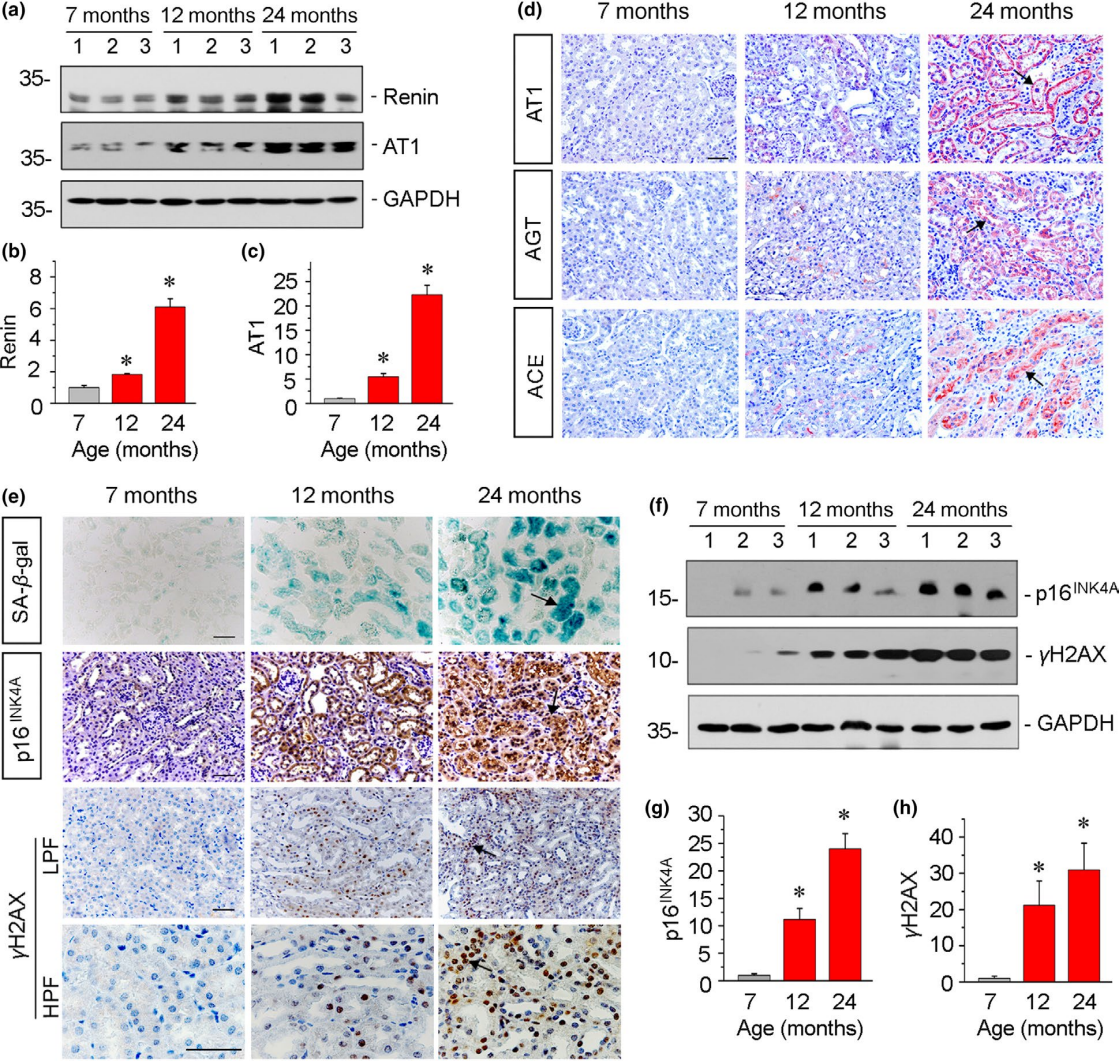
Renin–angiotensin system activity and cellular senescence are increased in aging kidneys. (a–c) Western blot analyses show the expression of renin and AT1. Graphical representations of (b) renin and (c) AT1 protein expression levels in different groups are presented. *p < .05 versus 7‐month‐old mice (*n* = 5–6). (d) Representative micrographs show the expression of AT1, AGT, and ACE in kidneys. Arrows indicate positive staining. Scale bar, 50 μm. (e) Representative staining micrographs show the expression of SA‐β‐gal activity, p16^INK4A^, and γH2AX in kidneys. Paraffin kidney sections were immunostained with an antibody against p16^INK4A^, which displays the expression in the nucleus and tubular cytoplasmic area. Paraffin sections were also stained for γH2AX, which displays nuclear location. Frozen kidney sections were stained for SA‐β‐gal activity, which appears as bright‐blue granular staining in the cytoplasm of tubular epithelial cells. Arrows indicate positive staining. Scale bar, 50 µm. LPF, low‐power field; HPF, high‐power field. (f–h) Western blot analyses show renal expression of p16^INK4A^ and γH2AX. Graphical representations of (g) p16^INK4A^ and (h) γH2AX protein expression levels in different groups are presented. *p < .05 versus 7‐month‐old mice (*n* = 5–6)

Our previous report shows RAS components are targets of Wnt/β‐catenin signaling (Zhou, Li, et al., 2015; Zhou, Mo, et al., 2015). Hence, we assessed the expression of RAS components. As shown in Figure 2a–c, the expression levels of renin and AT1 were significantly upregulated in 12‐month‐old mice and were even more so in 24‐month‐old mice. A similar result was observed when AT1 was tested by immunostaining. As shown in Figure 2d, AT1 was predominantly localized in tubular cells. We also examined the expression of angiotensinogen (AGT) and angiotensin‐converting enzyme (ACE), the other two important components of RAS. Similarly, they were upregulated in aged mice (Figure 2d). Consistently, a previous report shows that RAS activation is closely associated with aging (Kim et al., 2018). These results suggest the activation of the Wnt/β‐catenin/RAS axis plays a potential role in age‐related renal fibrosis.

Furthermore, we analyzed cellular senescence by investigating senescence‐associated β‐galactosidase (SA‐β‐gal) activity, p16^INK4A^, and γH2AX (Luo et al., 2018) in 7‐, 12‐, and 24‐month‐old mice. As shown in Figure 2e, these senescence markers were detected predominantly in tubular cells in aging kidneys. The similar results were observed when the protein expression levels of p16^INK4A^ and γH2AX were analyzed by Western blot analyses (Figure 2f–h).

### 2.2 Mitochondrial homeostasis is impaired in aging kidneys

**Figure 3:**
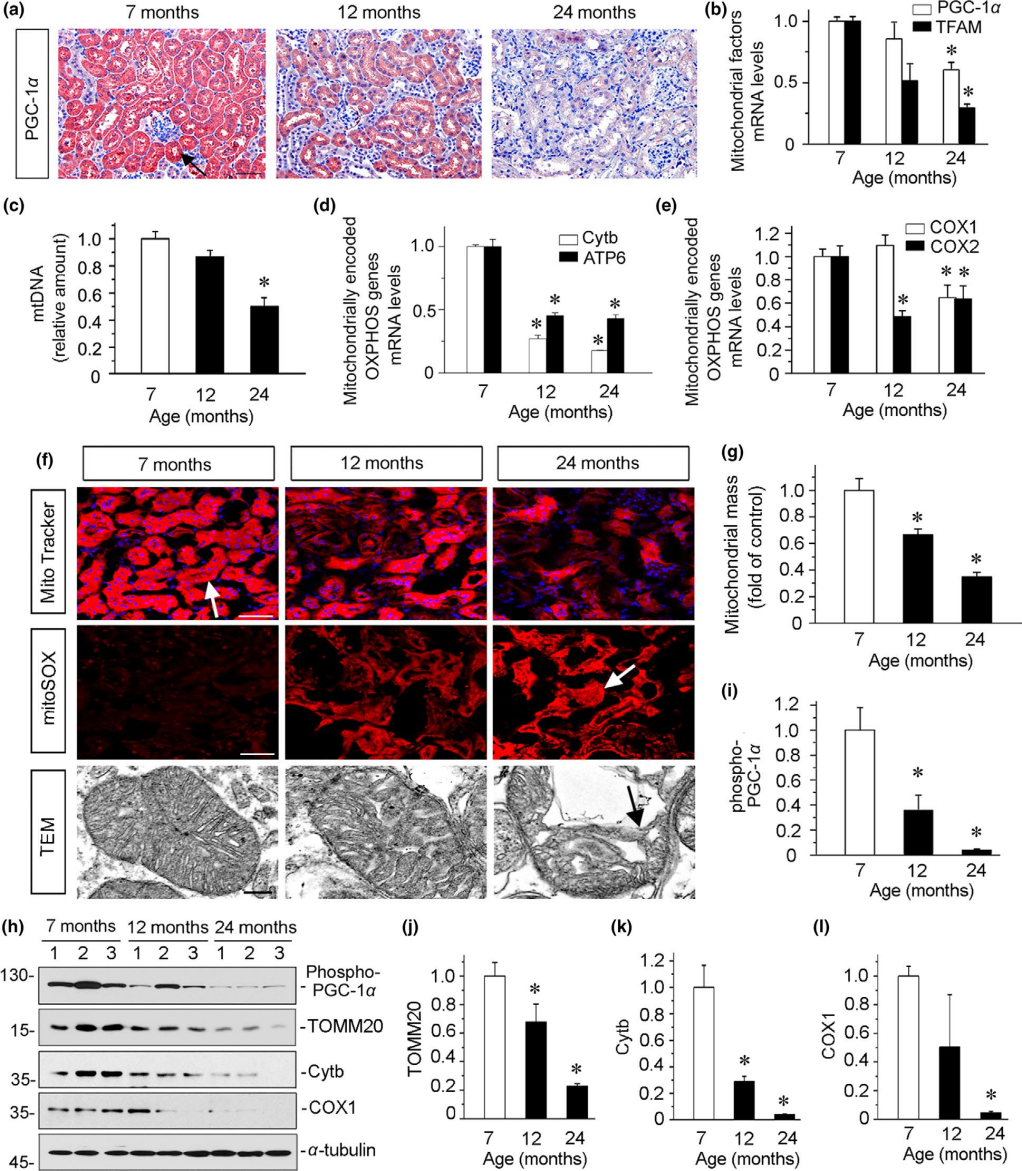
Mitochondrial homeostasis is impaired in aging kidneys. (a) Representative micrographs show renal expression of PGC‐1α. Arrow indicates positive staining. Scale bar, 50 μm. (b) Graphical representations of the relative abundance of mitochondrial factors mRNA in kidneys. PGC‐1α and TFAM mRNA levels in different groups were assessed by real‐time PCR. *p < .05 versus 7‐month‐old mice (*n* = 5–6). (c) Mitochondrial DNA (mtDNA) content in kidneys was analyzed. Total DNA was extracted and amplified with the primer of mitochondrial cytochrome *c* oxidase 2 (COX2) and normalized to ribosomal protein s18 (RSP18). *p < .05 versus 7‐month‐old mice (*n* = 5–6). (d–e) Graphical representations of the relative abundance of mitochondrial‐encoded OXPHOS genes are presented. Cytb, ATP6, COX1, and COX2 mRNA levels in different groups were assessed by real‐time PCR. *p < .05 versus 7‐month‐old mice (*n* = 5–6). (f) Representative fluorescence and transmission electron microscopy micrographs show mitochondrial mass, ROS production, and ultrastructure morphology. The frozen kidney sections were stained with MitoTracker deep red (3 μm) and mitoSOX (3.5 μm) probes. DAPI (4′,6‐diamidino‐2‐phenylindole) was used to stain the nuclei (blue). Ultrathin kidney sections were studied using a transmission electron microscope. For MitoTracker and mitoSOX staining, arrows indicate positive staining; For TEM analyses, arrow indicates abnormal characteristics of swollen shape and fragmented cristae in mitochondria. Scale bar, 50 μm for MitoTracker and mitoSOX staining; 1 μm for electron microscope micrographs. TEM, transmission electron microscopy. (g) Quantification of mitochondrial mass in kidneys is shown. Mitochondrial mass was determined by the rate of MitoTracker deep red fluorescence intensity normalized to DAPI. *p < .05 versus 7‐month‐old mice (*n* = 5–6). (h–l) Representative (h) Western blots and graphical representations of (i) phospho‐PGC‐1α, (j) TOMM20, (k) Cytb, and (l) COX1 protein expression levels in different groups are presented. *p < .05 versus 7‐month‐old mice (*n* = 5–6)

We first investigated the mitochondrial biogenesis‐related transcription factors peroxisome proliferator‐activated receptor‐C coactivator‐1α (PGC‐1α) and transcription factor A, mitochondrial (TFAM), two master regulators governing mitochondrial biogenesis (Dorn, Vega, & Kelly, 2015; Gomes et al., 2013). As shown in Figure 3a, PGC‐1α was downregulated with age, especially by 24 months of age. Furthermore, we assessed the mRNA expression levels of PGC‐1α and TFAM. Compared with the expression of normal adults (7 months of age), PGC‐1α and TFAM decreased by 12 months of age and significantly downregulated by 24 months of age (Figure 3b). We then analyzed mtDNA content by investigating cytochrome *c* oxidase subunit 2 (COX2), an important mitochondrially encoded oxidative phosphorylation (OXPHOS) complex IV subunit to transfer electrons from cytochrome *c* to oxygen (Gomes et al., 2013). As shown in Figure 3c, mtDNA content declined in 12‐month‐old mice and was substantially lower in mice at 24 months of age. Next, we tested the mRNA expression levels of mtDNA‐encoded OXPHOS complex III subunit cytochrome *b* (Cytb), complex V subunit ATP synthase 6 (ATP6), and complex IV subunits cytochrome *c* oxidase 1 (COX1) and cytochrome *c* oxidase 2 (COX2). As shown in Figure 3d,e, nearly all of these mitochondrially encoded OXPHOS genes significantly decreased in 12‐month‐old mice and even more declined in 24‐month‐old mice.

We further analyzed renal mitochondrial mass by MitoTracker deep red staining, mitochondrial ROS by mitoSOX staining, and the ultrastructural analysis of mitochondria using electron microscopy. As shown in Figure 3f,g, mitochondrial mass was significantly lower in 12‐month‐old mice and even less in 24‐month‐old mice. The mitoSOX staining showed that mitochondrial ROS production increased in aging kidneys progressively over the timepoints assessed (Figure 3f). The normal round or rod‐shaped mitochondria and regular arrangement of mitochondrial cristae were detected in 7‐month‐old mice, however, swollen mitochondria with disorganized and fragmented cristae appeared in 12‐month‐old mice, and yet more by 24 months of age (Figure 3f). Next, we assessed phosphorylated PGC‐1α (p‐PGC‐1α), the active form of PGC‐1α (Dorn et al., 2015), the outer mitochondrial membrane protein translocase of outer mitochondrial membrane 20 (TOMM20), Cytb, and COX1. As shown in Figure 3h–l, the expression levels of these proteins were significantly downregulated with age.

### 2.3 Klotho ameliorates age‐related renal fibrosis in a mouse model of accelerated aging

To further elucidate the correlation between Wnt/β‐catenin signaling and mitochondrial dysfunction in age‐related renal fibrosis, we performed a study in C57BL/6 mice by injection of d‐galactose (d‐gal), an accelerated aging model that has similarities to natural aging in rodents (Bo‐Htay, Palee, Apaijai, Chattipakorn, & Chattipakorn, 2018). The plasmid pV5‐sKlotho for secreted Klotho, an anti‐aging protein that serves as an endogenous Wnt antagonist (Zhou et al., 2013), was injected by hydrodynamic‐based gene delivery, an approach that is commonly used to ectopically express proteins in liver and kidneys (He et al., 2010; Zhou, Li, et al., 2015; Zhou et al., 2013; Zhou, Mo, et al., 2015). Delivery of naked pV5‐sKlotho plasmid led to a substantial expression of secreted Klotho protein in kidneys and liver 24 hr after intravenous injection (Figure S3a). This finding is consistent with results from our previous publication (Zhou et al., 2013).

**Figure 4:**
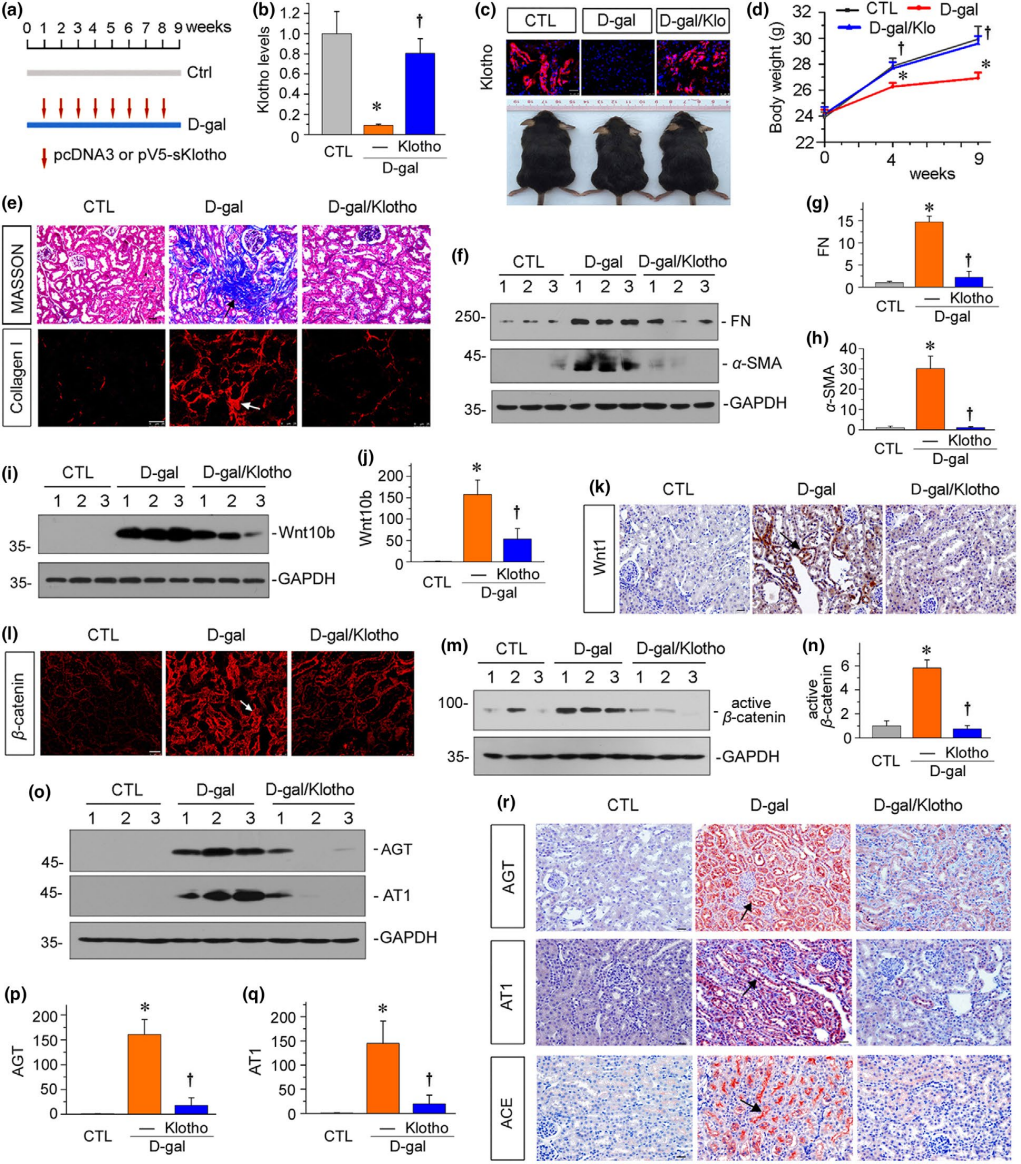
Klotho ameliorates age‐related renal fibrosis in vivo. (a) Experimental design. Red arrows indicate the injections of pcDNA3 or pV5‐sKlotho plasmid. Blue line indicates the timing of d‐gal treatment. (b) Graphical representation of Klotho protein expression levels in different groups. *p < .05 versus control mice; †*p* < .05 versus d‐gal‐treated mice (*n* = 5–6). (c) Representative immunofluorescence micrographs show Klotho expression in kidneys. Scale bar, 25 µm. And representative photographs show Klotho reversed d‐gal‐induced growth retardation. Klo, Klotho. (d) Graphical representation shows Klotho increased body weight gain in d‐gal‐treated mice. *p < .05 versus control mice; †*p* < .05 versus d‐gal‐treated mice (*n* = 5–6). (e) Representative micrographs show Masson trichrome staining and collagen I expression in kidneys. Paraffin sections were used for Masson trichrome staining. Frozen sections were used for immunofluorescence staining of collagen I. Arrows indicate positive staining. Scale bar, 25 µm. (f–h) Representative (f) Western blots and graphical representations of (g) fibronectin and (h) α‐SMA are presented. FN, fibronectin. *p < .05 versus control mice; †*p* < .05 versus d‐gal‐treated mice (*n* = 5–6). (i–j) Representative (i) Western blots and graphical representation of (j) Wnt10b in kidneys are presented. *p < .05 versus control mice; †*p* < .05 versus d‐gal‐treated mice (*n* = 5–6). (k–l) Representative micrographs show renal expression of (k) Wnt1 and (l) β‐catenin. Paraffin kidney sections were immunostained with an antibody against Wnt1. Frozen kidney sections were immunostained with an antibody against β‐catenin. Arrows indicate positive staining. Scale bar, 25 µm. (m, n) Representative (m) Western blots and graphical representation of (n) active β‐catenin protein expression in kidneys are presented. *p < .05 versus control mice; †*p* < .05 versus d‐gal‐treated mice (*n* = 5–6). (o–q) Representative (o) Western blots and graphical representations of (p) AGT and (q) AT1 protein expression levels in different groups are presented. *p < .05 versus control mice; †*p* < .05 versus d‐gal‐treated mice (*n* = 5–6). (r) Representative micrographs show renal expression of AGT, AT1, and ACE. Paraffin kidney sections were used for immunohistochemistry staining. Arrows indicate positive staining. Scale bar, 25 µm

The plasmid pV5‐sKlotho or empty vector (pcDNA3) was injected through the tail vein every week in d‐gal‐treated mice (Figure 4a). Western blot and immunofluorescence analyses revealed that renal Klotho protein expression was induced after injection of Klotho expression plasmid (Figure 4b,c). Compared with the slow growth of body weight in d‐gal‐treated mice, in vivo expression of exogenous Klotho significantly promoted body weight gain (Figure 4c,d). We then assessed renal fibrosis. As shown in Figure 4e, Masson trichrome staining and immunofluorescence staining for collagen I revealed that Klotho attenuated renal fibrotic lesions in d‐gal‐treated mice. Consistent with this observation, Klotho significantly decreased the expression of fibronectin and α‐SMA (Figure 4f–h). Next, we assessed the expression of Wnt10b and Wnt1. As shown in Figure 4i,j, the administration of d‐gal significantly induced the expression of Wnt10b. However, the expression of Wnt10b was largely inhibited by injection of exogenous Klotho plasmid. A similar result was seen when Wnt1 was tested by immunostaining (Figure 4k). To testify whether Klotho could inhibit Wnt expression, we performed in vitro studies. In HKC‐8 cells, Klotho bound to Wnt10b and inhibited Wnt10b‐induced activation of β‐catenin in d‐gal‐treated cells, but had no effects on the mRNA and protein levels of Wnt10b (Figure S4). This is consistent with our previous publication (Zhou et al., Journal of the American Society of Nephrology 2013), in that Klotho binds to Wnt1, and Klotho does not inhibit Wnt1 expression in renal tubular cells. Therefore, we speculate that the decrease in Wnt protein expression lies in the recovery from diseases. Consistent with these findings, the activation of β‐catenin, the downstream transcriptional regulator of Wnt signaling, was induced by d‐gal but significantly inhibited by ectopic expression of Klotho (Figure 4l–n). We then analyzed the expression of RAS proteins. As shown in Figure 4o–q, the expression levels of AGT and AT1 were significantly induced by administration of d‐gal. However, injection of Klotho plasmid largely blocked these effects. Similar results were observed when AGT, AT1, and ACE were assessed by immunostaining (Figure 4r).

### 2.4 Klotho protects mitochondrial functions in kidneys in an accelerated aging mouse model

**Figure 5:**
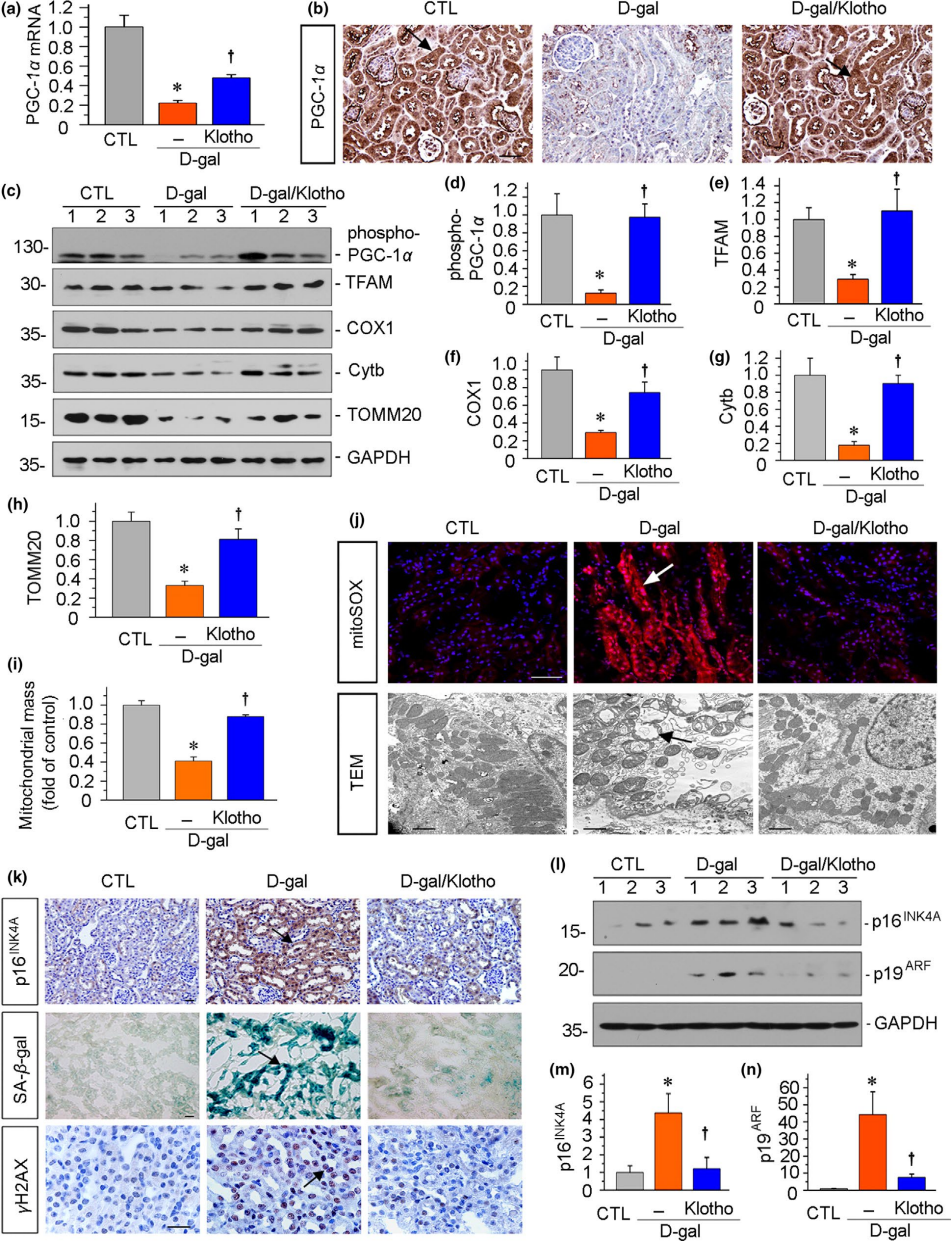
Ectopic expression of Klotho protects renal mitochondrial homeostasis and inhibits tubular senescence in an accelerated aging mouse model. (a) Renal expression of PGC‐1α mRNA in different groups was assessed by real‐time PCR. *p < .05 versus control mice; †*p* < .05 versus d‐gal‐treated mice (*n* = 5–6). (b) Representative micrographs show renal expression of PGC‐1α. Paraffin sections were immunostained for PGC‐1α. Arrows indicate positive staining. Scale bar, 50 µm. (c–h) Representative (c) Western blots and graphical representations of (d) phospho‐PGC‐1α, (e) TFAM, (f) COX1, (g) Cytb, and (h) TOMM20 protein expression levels in different groups are presented. *p < .05 versus control mice; †*p* < .05 versus d‐gal‐treated mice (*n* = 5–6). (i) Quantification of renal mitochondrial mass is shown. Mitochondrial mass was determined by the fluorescence intensity of MitoTracker deep red staining normalized to DAPI. *p < .05 versus control mice; †*p* < .05 versus d‐gal‐treated mice (*n* = 5–6). (j) Representative micrographs show mitochondrial ROS production and ultrastructure. They were assessed by mitoSOX staining and electron microscopy analyses, respectively. The administration of d‐gal induced mitochondrial ROS production, mitochondrial swelling, and cristae disorganization in renal tubular cells. Arrows indicate positive staining. Ectopic expression of Klotho inhibited mitochondrial ROS production and preserved normal structure of mitochondria. Scale bar in mitoSOX staining, 50 μm. Scale bar in TEM, 1 μm. TEM, transmission electron microscopy. (k) Representative staining micrographs show renal expression of p16^INK4A^, SA‐β‐gal activity, and γH2AX. Paraffin kidney sections were stained with antibodies against p16^INK4A^ and γH2AX. Frozen kidney sections were stained for SA‐β‐gal activity. Arrows indicate positive staining. Scale bar, 25 μm. (l–n) Representative (l) Western blots and graphical representations of (m) p16^INK4A^ and (n) p19^ARF^ protein expression levels in kidneys are presented. *p < .05 versus control mice; †*p* < .05 versus d‐gal‐treated mice (*n* = 5–6)

We next investigated the protective role of Klotho in age‐related mitochondrial dysfunction. First, PGC‐1α mRNA and protein expression levels were assessed by real‐time PCR and immunostaining. As shown in Figure 5a,b d‐gal significantly reduced PGC‐1α mRNA and protein expression. However, injection of Klotho plasmid significantly restored the expression of PGC‐1α in d‐gal‐treated mice. We then analyzed the expression of p‐PGC‐1α, TFAM, COX1, Cytb, and TOMM20 by Western blot analyses. As shown in Figure 5c–h, ectopic expression of Klotho significantly reversed these proteins in d‐gal‐treated mice, suggesting a protective role of inhibition of Wnt/β‐catenin in mitochondrial homeostasis. We then checked mitochondrial mass, mitochondrial ROS, and the ultrastructure of mitochondria. As shown in Figure 5i,j, Klotho significantly reversed the loss of mitochondrial mass and inhibited the production of mitochondrial ROS in mice injected with d‐gal. Furthermore, transmission electron microscopy analysis showed d‐gal induced pronounced changes in tubular cell mitochondria with mitochondrial swelling and cristae disorganization. Notably, injection of Klotho plasmid preserved a normal structure of mitochondria (Figure 5j).

We next examined cellular senescence. As shown in Figure 5k, treatment with d‐gal markedly induced the expression of p16^INK4A^, SA‐β‐gal activity, and γH2AX nuclear foci, the three key markers of aging. It is notable that the administration of d‐gal dramatically induced cellular senescence (Figure 5k). The reason behind this maybe that chronic d‐gal exposure augments senescence‐related signals by reacting with the free amines of amino acids to form advanced glycation end products (AGE) (Bo‐Htay et al., 2018), a strong trigger of oxidative stress and inflammation, the two main mechanisms of aging (Feng et al., 2018; Zhou et al., 2012). We then checked the effect of Klotho. Consistent with the protection of mitochondrial functions by Klotho, cellular senescence was greatly inhibited by the administration of Klotho plasmid (Figure 5k). Similar results were seen when p16^INK4A^ and p19^ARF^ proteins were assessed by Western blot analyses (Figure 5l–n).

### 2.5 DKK1 and mitoquinone (mitoQ) preserve mitochondrial functions and inhibit cellular senescence in an established aging model

**Figure 6:**
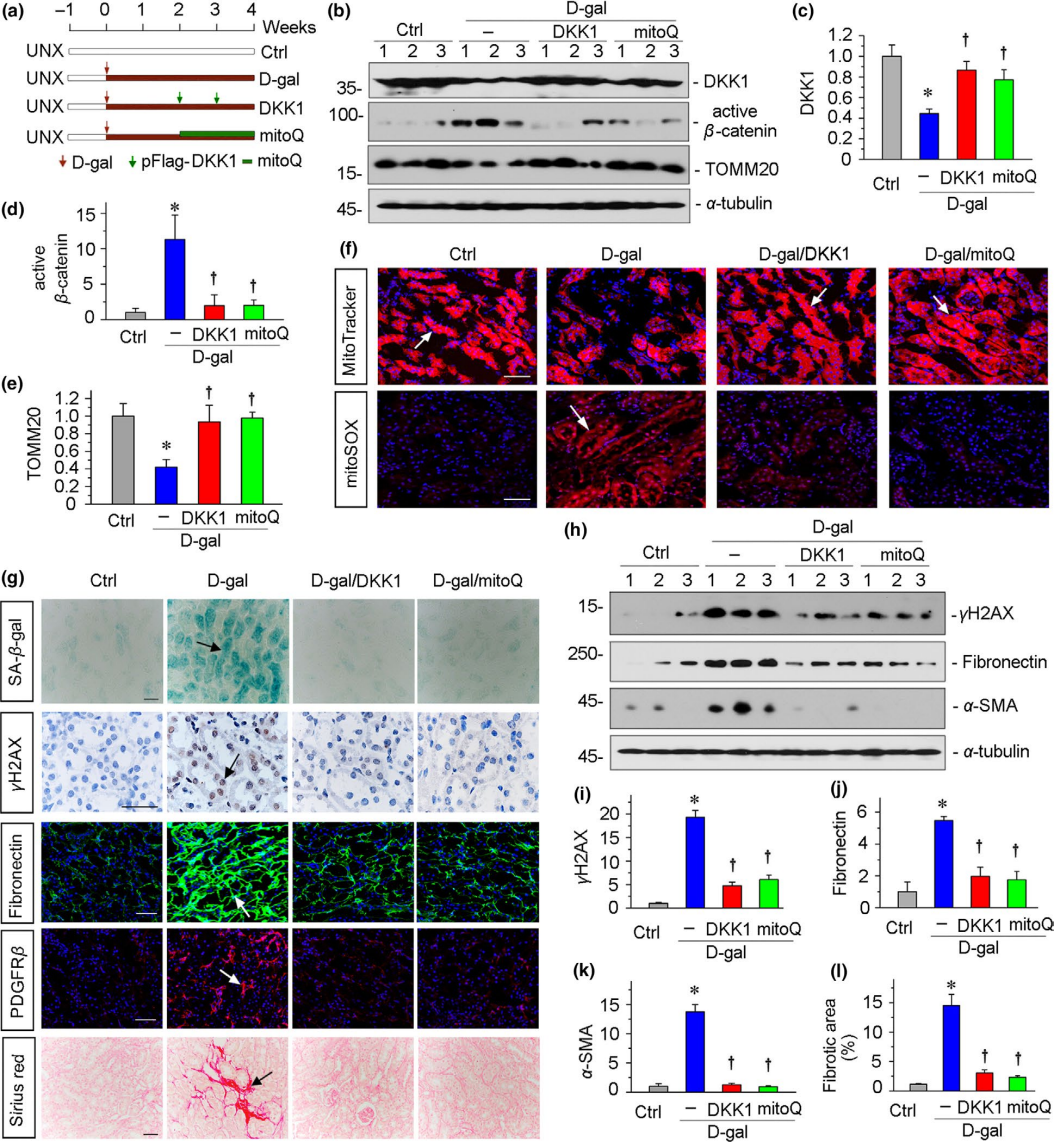
DKK1 and mitoQ preserve mitochondrial functions and inhibit cellular senescence in an established aging model. (a) Experimental design. Red arrows indicate the starting point of d‐gal injection. Green arrows indicate the injections of pFlag‐DKK1 plasmid. Green bar indicates treatment with mitoQ. (b–e) Representative (b) Western blots and graphical representations of (c) DKK1, (d) active β‐catenin, and (e) TOMM20 are presented. *p < .05 versus control mice; †*p* < .05 versus d‐gal‐treated mice (*n* = 5–6). (f) Representative MitoTracker and mitoSOX staining micrographs show the mitochondrial mass loss and ROS production induced by d‐gal were blocked by DKK1 or mitoQ. Arrows indicate positive staining. Scale bar, 50 µm. (g) Representative staining micrographs show renal expression of SA‐β‐gal activity, γH2AX, fibronectin, PDGFRβ, and Sirius red staining. Frozen kidney sections were stained for SA‐β‐gal activity, fibronectin, and PDGFRβ. Paraffin sections were immunostained with an antibody against γH2AX or performed with Sirius red staining. Arrows indicate positive staining. Scale bar, 50 μm. (h–k) Representative (h) Western blots and graphical representations of (i) γH2AX, (j) fibronectin, and (k) α‐SMA protein expression levels in kidneys are presented. *p < .05 versus control mice; †*p* < .05 versus d‐gal‐treated mice (*n* = 5–6). (l) Quantitative determination of renal fibrotic lesions in different groups. *p < .05 versus control mice; †*p* < .05 versus d‐gal‐treated mice (*n* = 5–6)

To further explore the role of Wnt/β‐catenin signaling in mitochondrial dysfunction and the association with age‐related renal fibrosis, we injected mice with the expression plasmid of DKK1, a more specific Wnt inhibitor (He et al., 2010), or the mitochondria‐targeted antioxidant mitoQ (Xiao et al., 2017) after 2 weeks of daily injection of d‐gal in a mouse model of uninephrectomy (Figure 6a), an established age‐associated kidney fibrosis model in which mitochondrial dysfunction and cellular senescence had already occurred (Figure S5). In human kidneys, with even healthy aging, there is a cortical volume loss accompanied by underlying nephrosclerosis with nephron loss, tubular hypertrophy, and diverticuli (Hommos et al., 2017). Hence, we thought that the administration of d‐gal in the uninephrectomized mice could be more relevant for age‐related renal fibrosis. The efficacy of DKK1 gene delivery was observed by Western blot analyses in liver and kidneys 24 hr after injection of DKK1 expression plasmid (Figure S3b). Other mice were sacrificed after 4 weeks of injection of d‐gal (Figure 6a). We first assessed the expression of DKK1 and found it was downregulated after administration of d‐gal, but greatly preserved by ectopic expression of DKK1 or administration of mitoQ (Figure 6b,c and Figure S6). Furthermore, the expression of active β‐catenin was inhibited by treatment with DKK1 or mitoQ in d‐gal‐treated mice (Figure 6b,d). We next analyzed mitochondrial mass by assessing TOMM20 protein and mitochondrial fluorescence intensity by staining with MitoTracker deep red probe. As shown in Figure 6b,e,f, the administration of d‐gal significantly decreased the expression of TOMM20 and diminished the mitochondrial fluorescence intensity, suggesting a loss of mitochondrial mass. However, treatment with DKK1 or mitoQ largely preserved mitochondrial mass. Moreover, the mitoSOX probe staining showed d‐gal‐induced mitochondrial ROS production was also greatly inhibited by ectopic expression of DKK1 or administration of mitoQ (Figure 6f). We then tested cellular senescence and renal fibrosis. As shown in Figure 6g, SA‐β‐gal activity and γH2AX staining showed administration of d‐gal induced tubular cell senescence, but treatment with DKK1 or mitoQ largely inhibited it. A similar result was observed when γH2AX protein expression was analyzed by Western blot (Figure 6h,i). We further assessed renal fibrosis. As shown in Figure 6g, the immunofluorescence staining for fibronectin and PDGFRβ and Sirius red staining indicated that treatment with DKK1 or mitoQ could ameliorate renal fibrosis in d‐gal‐treated mice. Similar results were observed when fibronectin and α‐SMA proteins were analyzed by Western blot (Figure 6h,j,k). The quantification of renal fibrosis also demonstrated DKK1 or mitoQ mitigated age‐related fibrotic lesions in an established disease state (Figure 6l).

### 2.6 Wnt/β‐catenin/RAS axis plays a central role in mitochondrial dysfunction and cellular senescence

**Figure 7:**
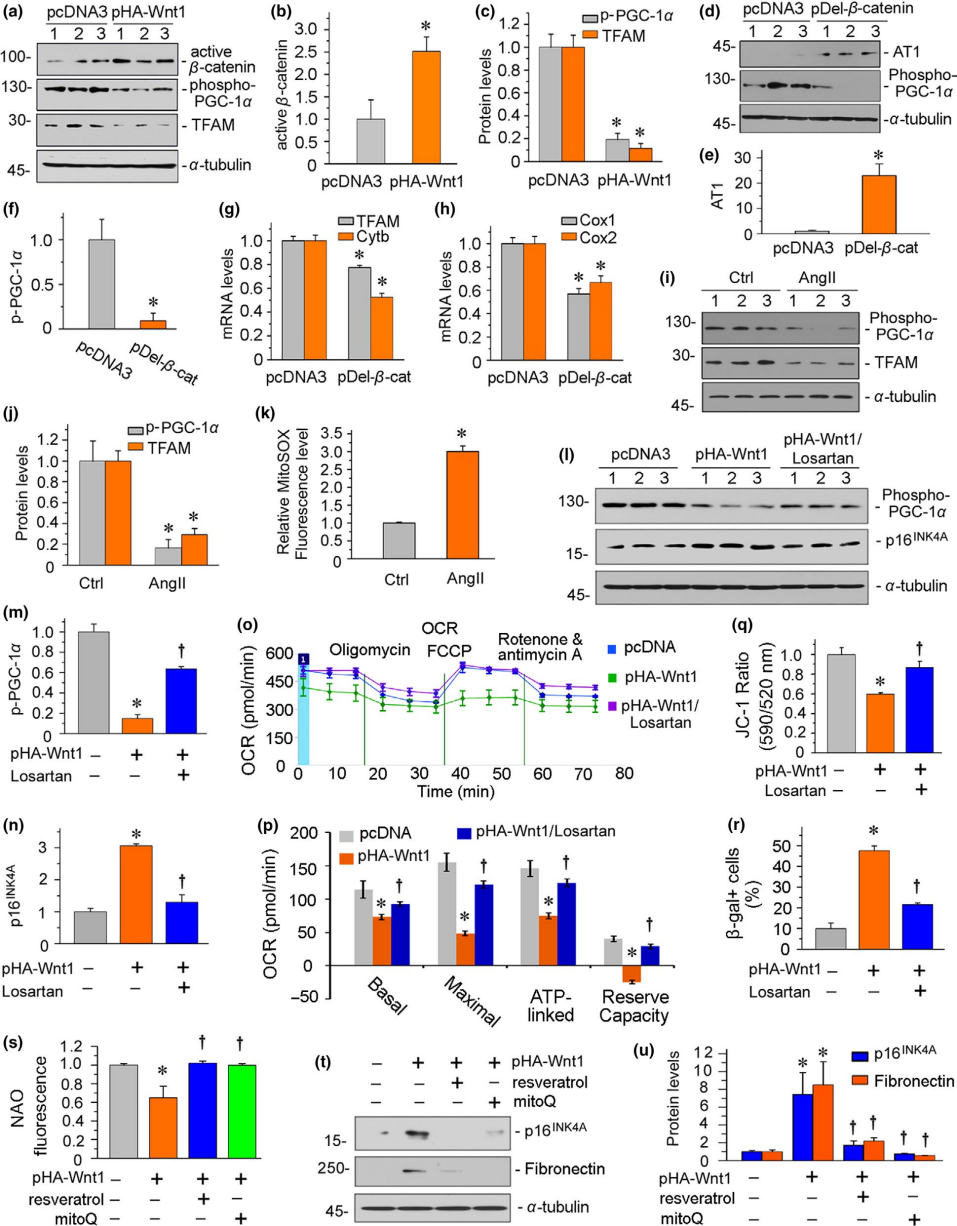
Wnt/β‐catenin/RAS axis plays a central role in mitochondrial dysfunction and cellular senescence in renal tubular cells. (a–c) Representative (a) Western blots and graphical representations of (b) active β‐catenin, (c) phospho‐PGC‐1α, and TFAM protein expression levels in different groups are presented. HKC‐8 cells were transfected with empty vector (pcDNA3) or Wnt1 expression plasmid (pHA‐Wnt1) for 24 hr. Whole‐cell lysates were analyzed by Western blot. *p < .05 versus pcDNA3 group (*n* = 3). (d–f) Representative (d) Western blots and graphical representations of (e) AT1 and (f) phospho‐PGC‐1α are presented. HKC‐8 cells were transfected with empty vector (pcDNA3) or β‐catenin expression plasmid (pDel‐β‐catenin) for 24 hr. Whole‐cell lysates were analyzed by Western blot. *p < .05 versus pcDNA3 group (*n* = 3). (g–h) Graphical representations of the relative mRNA abundance of mitochondrial‐encoded OXPHOS genes in two groups are presented. The mRNA levels of (g) TFAM and Cytb, as well as (h) COX1 and COX2 in two groups, were assessed. HKC‐8 cells were transfected with empty vector (pcDNA3) or β‐catenin expression plasmid (pDel‐β‐catenin) for 24 hr. Total RNA was extracted and analyzed by quantitative real‐time PCR. *p < .05 versus pcDNA3 group (*n* = 3). (i, j) Representative (i) Western blots and graphical representations of (j) phospho‐PGC‐1α and TFAM are presented. HKC‐8 cells were treated with AngII (10 nM) for 24 hr. Whole‐cell lysates were analyzed by Western blot. *p < .05 versus the control group (*n* = 3). Ctrl, control. (k) Graphical representation shows AngII significantly induced mitochondrial superoxide production in HKC‐8 cells. HKC‐8 cells were treated with AngII (10 nM) for 12 hr and then incubated with mitochondrial superoxide indicator mitoSOX™ Red (5 μM) as manufacturer's instruction. The fluorescence was analyzed by flow cytometry. *p < .05 versus the control group (*n* = 3). Ctrl, control. (l–n) Representative (l) Western blots and graphical representations of (m) phospho‐PGC‐1α and (n) p16^INK4A^ are presented. HKC‐8 cells were pretreated with losartan (10 μM) for 1 hr and then transfected with Wnt1 expression plasmid for 24 hr. *p < .05 versus pcDNA3 group; †*p* < .05 versus pHA‐Wnt1 group (*n* = 3). (o) An analysis of O_2_ consumption in renal tubular cells. HKC‐8 cells were pretreated with losartan (10 μM) for 1 hr and then transfected with Wnt1 expression plasmid for 24 hr. OCR was first measured in approximately 3 × 10^4^ cells of each group under basal condition. Those cells then were sequentially added oligomycin (1 μM), FCCP (0.5 μM), rotenone (0.5 μM), and antimycin A (0.5 μM) to determine different parameters of mitochondrial functions according to the manufacturer's instructions. (p) Graphical representations of basal OCR, maximal OCR, ATP‐linked OCR, and reserve capacity. The average of four determinations for each group is shown. *p < .05 versus pcDNA3 group; †*p* < .05 versus pHA‐Wnt1 group (*n* = 4). (q) Graphical representation of mitochondrial membrane potential (MMP). MMP was detected by JC‐1 staining and analyzed by flow cytometry. After pretreatment with losartan (10 μM) for 1 hr, HKC‐8 cells were transfected with Wnt1 expression plasmid for 24 hr and then stained with JC‐1. The MMP is shown as the ratio of the fluorescence intensity at absorbance of 590 nm (JC‐1 aggregate) to 520 nm (JC‐1 monomer). *p < .05 versus pcDNA3 group; †*p* < .05 versus pHA‐Wnt1 group (*n* = 3). (r) Graphical representation of β‐galactosidase‐positive cells. After pretreatment with losartan (10 μM) for 1 hr, HKC‐8 cells were transfected with Wnt1 expression plasmid for 24 hr and then stained for β‐galactosidase activity. *p < .05 versus pcDNA3 group; †*p* < .05 versus pHA‐Wnt1 group (*n* = 3). (s) Graphical representation of mitochondrial mass determined by flow cytometry analysis of NAO fluorescence. The mean fluorescence intensities of 10,000 events were counted for each cell population. After pretreatment with losartan (10 μM) for 1 hr, HKC‐8 cells were transfected with Wnt1 expression plasmid for 24 hr and then stained with NAO (5 μM). *p < .05 versus pcDNA3 group; †*p* < .05 versus pHA‐Wnt1 group (*n* = 3). (t, u) Representative (t) Western blots and graphical representations of (u) p16^INK4A^ and fibronectin are presented. HKC‐8 cells were pretreated with resveratrol (50 μM) or mitoQ (100 nM) for 1 hr and then transfected with Wnt1 expression plasmid for 24 hr. Whole‐cell lysates were analyzed by Western blot. *p < .05 versus pcDNA3 group; †*p* < .05 versus pHA‐Wnt1 group (*n* = 3)

First, HKC‐8 cells, a renal proximal tubular cell line, were transfected with human Wnt1 expression plasmid (pHA‐Wnt1). As shown in Figure 7a–c, ectopic expression of Wnt1 significantly induced the activation of β‐catenin and decreased phosphorylation of PGC‐1α, the master regulator of mitochondrial biogenesis (Hickey et al., 2011). We then analyzed the expression of TFAM (transcription factor A, mitochondrial), a PGC‐1α‐controlled key transcription factor that regulates replication, transcription, and translation of mitochondrial OXPHOS genes (Gomes et al., 2013). As shown in Figure 7a,c, TFAM was greatly downregulated by ectopic expression of Wnt1. Furthermore, HKC‐8 cells were transfected with human β‐catenin expression plasmid (pDel‐β‐catenin). As shown in Figure 7d–f, overexpression of β‐catenin induced upregulation of AT1 and inhibited the expression of p‐PGC‐1α. Moreover, quantitative real‐time PCR results of TFAM and OXPHOS genes Cytb, COX1, and COX2 further confirmed the negative regulation on mitochondrial functions by β‐catenin (Figure 7g,h). To further determine the role of RAS activation in mitochondrial dysfunction, we first incubated HKC‐8 cells with angiotensin II (AngII). As shown in Figure 7i,j, the administration of AngII decreased the expression of p‐PGC‐1α and TFAM. Moreover, AngII triggered the production of mitochondrial ROS (Figure 7k). We then treated HKC‐8 cells with losartan, an AT1 receptor blocker. As shown in Figure 7l–n, losartan significantly inhibited Wnt1‐induced downregulation of p‐PGC‐1α and upregulation of p16^INK4A^. We next performed mitochondrial functional analysis of oxygen consumption rate (OCR) using the Seahorse assay. The basal OCR was determined as the OCR before oligomycin minus OCR after rotenone/antimycin A. The maximal OCR was determined as the OCR after FCCP (carbonyl cyanide 4‐(trifluoromethoxy) phenylhydrazone) treatment minus nonmitochondrial OCR. ATP‐linked OCR was determined as the OCR before oligomycin minus OCR after oligomycin treatment. The reserve capacity was defined as the difference between maximal OCR after FCCP treatment and basal OCR. Furthermore, the mitochondrial membrane potential (MMP) was assessed by JC‐1 staining (Figure S7a). As shown in Figure 7o–q, overexpression of Wnt1 significantly decreased the levels of oxygen consumption, ATP production, and MMP. Losartan nearly completely blocked these effects. Moreover, the FACS‐based β‐gal staining results further demonstrated losartan largely inhibited Wnt1‐induced cellular senescence (Figure 7r and Figure S7b). Similar results were observed when tubular cells were transfected with a β‐catenin expression plasmid (pDel‐β‐catenin) (Figure S8). These data suggest the mediating role of RAS activation in Wnt/β‐catenin‐induced mitochondrial dysfunction.

To further clarify the role of Wnt/β‐catenin in cellular senescence and its association with mitochondrial dysfunction, tubular cells were pretreated with resveratrol, a drug that activates SIRT1 and leads to activation of PGC‐1α (Lagouge et al., 2006), and the mitochondrial ROS scavenger mitoQ, then transfected with a Wnt1 expression plasmid. A FACS analysis of nonyl acridine orange (NAO) fluorescence showed that the loss of mitochondrial mass was induced by overexpression of Wnt1, but was blocked by the administration of resveratrol or mitoQ (Figure 7s and Figure S7c). Furthermore, the Western blot analyses of p16^INK4A^ and fibronectin showed that resveratrol and mitoQ protected against cellular senescence and fibrotic lesions (Figure 7t,u).

### 2.7 Wnt/β‐catenin/RAS axis mediates mitochondrial dysfunction and age‐related renal fibrosis in vitro

**Figure 8:**
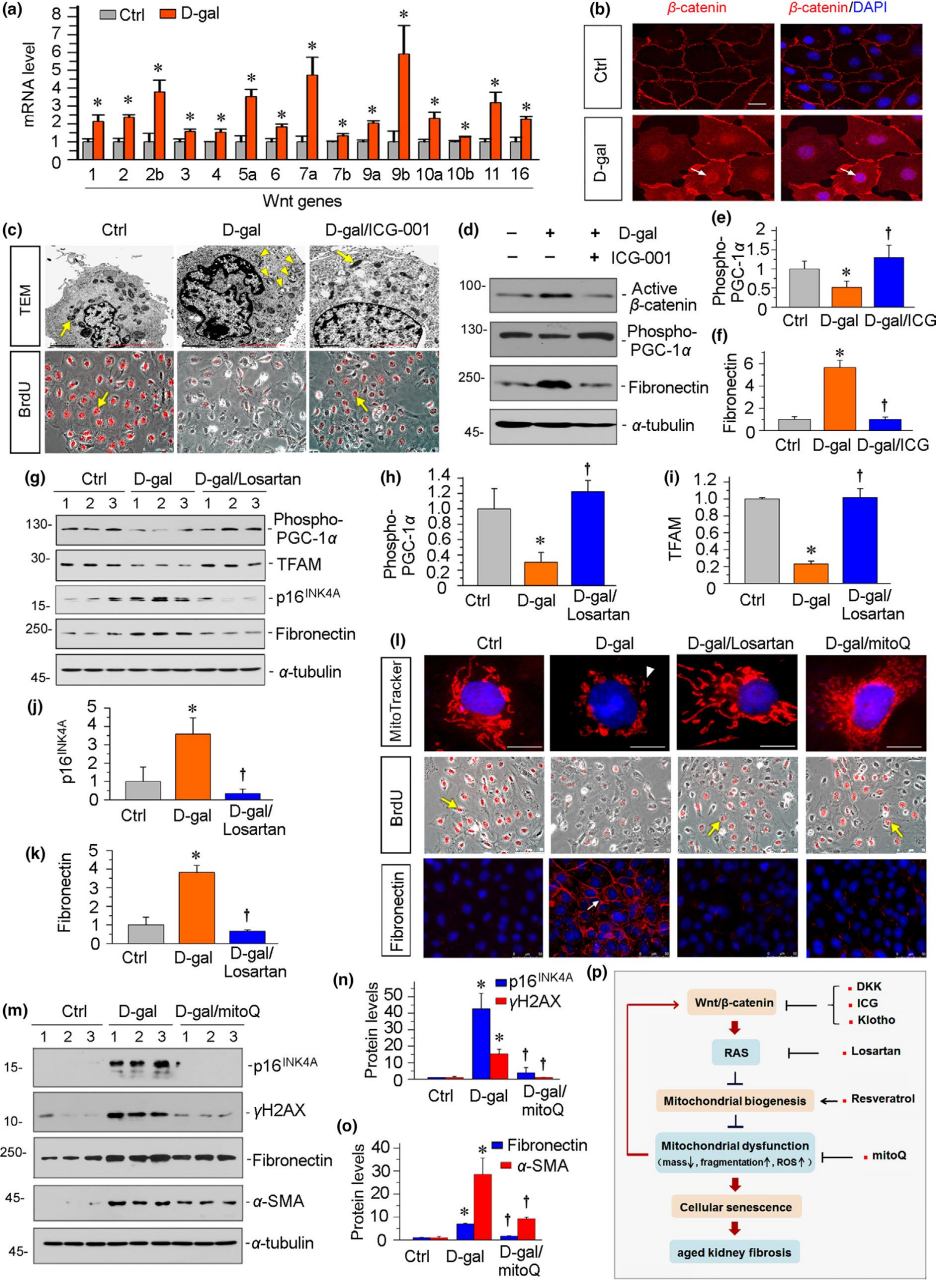
Wnt/β‐catenin mediates age‐related renal fibrosis in vitro. (a) Real‐time PCR results show d‐gal induced upregulation of multiple Wnt genes in cultured proximal tubular cell line (HKC‐8). HKC‐8 cells were treated with d‐gal (10 mg/ml) for 60 hr. Total RNA was extracted and analyzed for various Wnt mRNA expression levels. *p < .05 versus control group. Ctrl, control. (b) Representative immunofluorescence micrographs show d‐gal induced nuclear translocation of β‐catenin. HKC‐8 cells were treated with d‐gal (10 mg/ml) for 60 hr and then stained for β‐catenin (red) and DAPI (blue). Arrows indicate nuclear staining of β‐catenin. Scale bar, 20 μm. (c) Representative electron microscopy and BrdU incorporation assay micrographs show mitochondria and cell proliferation in renal tubular cells. HKC‐8 cells were pretreated with ICG‐001 (5 μm) for 1 hr and then treated with d‐gal (10 mg/ml) for 60 hr. BrdU (10 μM) was added for 12 hr before collection. For TEM analyses, arrows indicate healthy mitochondria, and arrowheads indicate abnormal‐shaped mitochondria. Scale bar, 2 μm; for BrdU incorporation assay, arrows indicate BrdU incorporation positive cells. Scale bar, 75 μm. TEM, transmission electron microscopy. (d–f) Representative (d) Western blots and graphical representations of (e) phospho‐PGC‐1α and (f) fibronectin are presented. HKC‐8 cells were pretreated with ICG‐001 (5 μm) for 1 hr and then treated with d‐gal (10 mg/ml) for 60 hr. *p < .05 versus control group; †*p* < .05 versus d‐gal group (*n* = 3). (g–k) Representative (g) Western blots and graphical representations of (h) phospho‐PGC‐1α, (i) TFAM, (j) p16^INK4A^, and (k) fibronectin are presented. HKC‐8 cells were pretreated with losartan (10 μM) for 1 hr and then treated with d‐gal (10 mg/ml) for 60 hr. *p < .05 versus control group; †*p* < .05 versus d‐gal group (*n* = 3). (l) Representative fluorescence micrographs show MitoTracker, BrdU, and fibronectin staining. After pretreatment with losartan (10 μM) or mitoQ (100 nM) for 1 hr, HKC‐8 cells were treated with d‐gal (10 mg/ml) for 60 hr. BrdU (10 μM) was added for 12 hr before collection. Cells were stained with MitoTracker deep red probe (300 nM) or antibodies against BrdU and fibronectin. Arrowhead indicates the loss of mitochondrial mass and the increase in fragmentation of cristae. Arrows indicate positive staining for BrdU‐positive cells and fibronectin. Scale bar, 10 μm for images of MitoTracker staining. (m–o) Representative (m) Western blots and graphical representations of (n) p16^INK4A^ and γH2AX, and (o) fibronectin and α‐SMA are presented. HKC‐8 cells were pretreated with mitoQ (100 nM) for 1 hr and then treated with d‐gal (10 mg/ml) for 60 hr. *p < .05 versus control group; †*p* < .05 versus d‐gal group (*n* = 3). (p) Schematic presentation depicts the potential mechanism by which Wnt/β‐catenin induces age‐related renal fibrosis. Wnt/β‐catenin signaling triggers the activation of RAS, thereby leading to the injury of mitochondrial biogenesis. This causes mitochondrial dysfunction with a loss of mass and increase in fragmentation and ROS production, which in turn induces tubular cell senescence and age‐related renal fibrosis. The mitochondrial dysfunction and activation of Wnt/β‐catenin signaling reciprocally induce each other. Multiple approaches, such as the inhibition of Wnt/β‐catenin by DKK1, ICG‐001, or Klotho, the blockade of RAS by losartan, the protection of mitochondrial biogenesis by resveratrol, and the mitochondria‐targeted antioxidant mitoQ could slow age‐related renal fibrosis

We then examined the effects on mitochondrial functions and cellular senescence of Wnt/β‐catenin signaling in d‐gal‐treated HKC‐8 cells. First, we tested the expression levels of all Wnts mRNA. Among 19 Wnts, 15 Wnts were significantly induced by treatment with d‐gal (Figure 8a). d‐Gal also triggered nuclear translocation of β‐catenin, the downstream effector of Wnt signaling (Figure 8b). To further clarify the role of Wnt/β‐catenin signaling, HKC‐8 cells were pretreated with ICG‐001, a small molecule that blocks β‐catenin‐mediated gene transcription in a CBP (cAMP‐responsive element binding [CREB]‐binding protein)‐dependent manner (Zhou, Li, et al., 2015; Zhou, Mo, et al., 2015). As shown in Figure 8c, treatment with d‐gal decreased the numbers of healthy mitochondria and induced morphological abnormalities in mitochondria. The administration of ICG‐001 largely reversed d‐gal‐induced mitochondrial damage. We then assessed cell proliferation and cellular senescence. The results of BrdU incorporation assay showed cell proliferation was inhibited by treatment with d‐gal, but restored by ICG‐001 to a remarkable degree (Figure 8c). We next analyzed the expression of p‐PGC‐1α and fibronectin. As shown in Figure 8d–f, treatment with d‐gal decreased the expression of p‐PGC‐1α and induced the expression of fibronectin. The administration of ICG‐001 significantly reversed these changes in d‐gal‐treated cells, suggesting a potential role of Wnt/β‐catenin signaling in mitochondrial dysfunction and aged‐related renal fibrosis.

Furthermore, losartan reversed d‐gal‐induced loss of p‐PGC‐1α and TFAM and inhibited the upregulation of p16^INK4A^ and fibronectin (Figure 8g–k). We then investigated mitochondria. As shown in Figure 8l, MitoTracker labeling fluorescence analyses showed mitochondrial mass loss and fragmentation of cristae were induced by treatment with d‐gal. However, the administration of losartan largely preserved normal characteristics of mitochondria. The BrdU incorporation assay and immunofluorescence staining for fibronectin showed that losartan inhibited the loss of cell proliferation ability and the increase in fibrogenesis induced by d‐gal (Figure 8l). We then explored the effects of mitoQ on d‐gal‐treated cells. As shown in Figure 8l, mitoQ restored mitochondrial mass and cell proliferation, and inhibited fibrotic lesions in d‐gal‐treated cells. Furthermore, mitoQ significantly inhibited d‐gal‐induced cellular senescence as detected by Western blot analyses of p16^INK4A^ and γH2AX (Figure 8m,n). Similarly, mitoQ blocked the expression of fibronectin and α‐SMA, two well‐known markers of renal fibrosis (Figure 8m,o). These results suggest that the Wnt/β‐catenin/RAS axis plays a central role in age‐related renal fibrosis and is associated with mitochondrial dysfunction. Taken together, as summarized in Figure 8p, it is concluded that Wnt/β‐catenin signaling induces RAS activation, which promotes cellular senescence and age‐related kidney fibrosis in association with mitochondrial dysfunction that stems from a loss of mitochondrial biogenesis.

### 2.8 Wnt/β‐catenin induced age‐related mitochondrial dysfunction and renal fibrosis in primary cultured tubular cells

**Figure 9:**
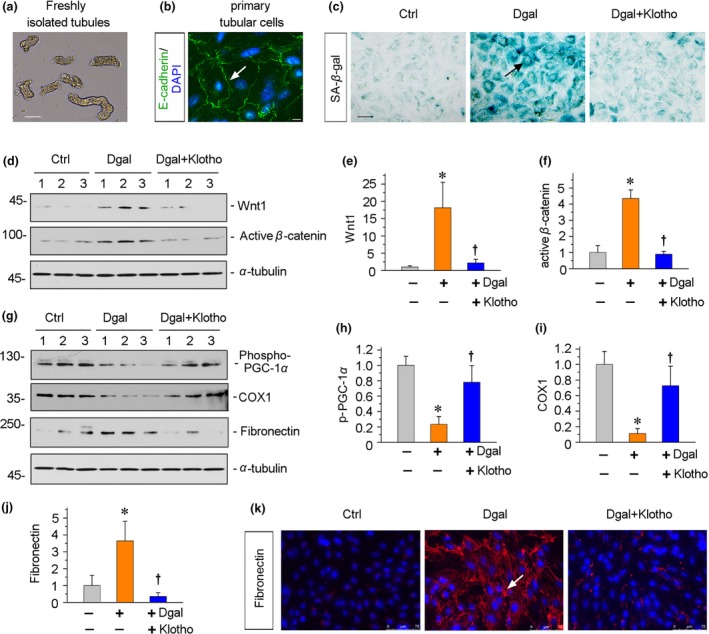
Wnt/β‐catenin induces age‐related renal fibrosis in primary cultured tubular cells. (a) Representative micrograph shows freshly isolated tubules. Renal tubules were isolated from mouse kidneys and cultivated for primary tubular cells. Scale bar, 50 μm. (b) Representative micrograph shows the staining of E‐cadherin (green) and DAPI (blue). Arrow indicates positive staining. Scale bar, 10 μm. (c) Representative micrographs show the staining of SA‐β‐gal activity. The primary cultured tubular cells were pretreated with Klotho (100 ng/ml) and then treated with d‐gal (10 mg/ml) for 60 hr. SA‐β‐gal activity was assessed according to the manufacturer's instructions. Arrow indicates positive staining. Scale bar, 50 μm. (d–f) Representative (d) Western blots and graphical representations of (e) Wnt1 and (f) active β‐catenin are presented. The primary tubular cells were treated as described. *p < .05 versus control group; †*p* < .05 versus d‐gal group (*n* = 3). (g–j) Representative (g) Western blots and graphical representations of (h) phospho‐PGC‐1α (p‐PGC‐1α), (i) COX1, and (j) fibronectin are presented. The primary tubular cells were treated as described. *p < .05 versus control group; †*p* < .05 versus d‐gal group (*n* = 3). (k) Representative fluorescence micrographs show the expression of fibronectin. Primary tubular cells were immunostained for fibronectin (red) and counterstained with DAPI (blue). Arrow indicates positive staining. Scale bar, 75 μm

We next cultured primary mouse renal tubular cells (Figure 9a,b). Incubation with d‐gal triggered an increase in SA‐β‐gal activity. Coculture with Klotho inhibited d‐gal‐induced cellular senescence (Figure 9c). Furthermore, Klotho significantly inhibited d‐gal‐induced upregulation of Wnt1 and activation of β‐catenin (Figure 9d–f). Consequently, Klotho restored the expression of p‐PGC‐1α and COX1, two important proteins in mitochondrial biogenesis and OXPHOS processes (Gomes et al., 2013) (Figure 9g–i). And Klotho substantially inhibited d‐gal‐induced upregulation of fibronectin (Figure 9g,j,k). These results further provide striking evidences that Wnt/β‐catenin signaling plays a key role in age‐related mitochondrial dysfunction and renal fibrotic lesions.

## 3 DISCUSSION

Chronic kidney disease has a high prevalence in aged people (Bruck et al., 2016; Minutolo et al., 2015), suggesting that aging plays an important role in the pathogenesis of CKD. Moreover, CKD exhibits many features of aging, including glomerular sclerosis and interstitial fibrosis, loss of Klotho, local RAS activation, increased oxidative stress, and persistent inflammation. These findings indicate that CKD can be viewed through the lens of premature aging (Hommos et al., 2017; Stenvinkel & Larsson, 2013). In an early stage of CKD, the senescent cells have accumulated in patients even with mild proteinuria (Verzola et al., 2008). Accelerated senescence and fibrosis are of mutual promotion, which leads to more advanced pathological features in the fragile kidneys (Bobkova et al., 2015; Luo et al., 2018). An evaluation of the underlying mechanisms of age‐related renal fibrosis can provide important therapeutic targets to treat CKD.

The mitochondrion is the major organelle that supplies energy and maintains cellular homeostasis to keep essential organ functions. Notably, defects in the electron transport chain would promote electron leakage to form superoxide radicals, which play a key role in triggering cellular senescence and accelerating aging (Barja, 2013). Hence, a better understanding of mitochondrial dysfunction would be of great significance to ameliorate aging.

Wnt/β‐catenin signaling is a developmental signaling pathway that is silent in normal adult kidneys. It is reactivated in various forms of CKD (He et al., 2009; Zhou et al., 2013), and plays a key role in driving CKD progression (Xiao et al., 2016). Among the multiple downstream targets of Wnt/β‐catenin signaling, the RAS was previously identified and reported by our group (Zhou, Li, et al., 2015; Zhou, Mo, et al., 2015). The findings indicate that Wnt/β‐catenin signaling is a master regulator of the RAS. Inhibition of Wnt/β‐catenin could simultaneously block multiple RAS genes. This certainly provides a more effective RAS blockade against aldosterone breakthrough as compared with inhibition by an ACE inhibitor (ACEI) or angiotensin receptor blocker (ARB) (Floege, 2015). It is noteworthy that angiotensin II blockade provides anti‐aging benefits (de Cavanagh, Inserra, & Ferder, 2011); hence, a more powerful RAS blockade would be of great importance. However, whether Wnt/β‐catenin signaling could promote age‐related renal fibrosis and its impairment of mitochondrial functions remains unknown. In this study, we showed that Wnt/β‐catenin/RAS signaling has a decisive role in driving mitochondrial dysfunction, and inhibition of Wnt/β‐catenin signaling effectively prevents age‐related renal fibrosis.

Compared with the low expression of Wnt/β‐catenin signaling and RAS activity in adults, they are greatly induced in elderly mice, with a concomitant development of renal fibrosis (Figures 1 and 2, Figures S1 and S2). In addition, mitochondrial biogenesis and OXPHOS functions are impaired during aging (Figure 3). These suggest an intimate role of Wnt/β‐catenin in age‐related mitochondrial dysfunction and renal fibrosis. In vivo and in vitro studies revealed that inhibition of Wnt/β‐catenin by Klotho significantly preserves mitochondrial functions and restrains the accelerated aging in kidneys (Figures 4 and 5). Furthermore, the direct induction of mitochondrial mass loss and malfunction, and the promotion of β‐galactosidase‐positive cells by overexpression of Wnt1 and β‐catenin in cultured tubular cells, provides striking evidences that the activation of Wnt/β‐catenin signaling impairs mitochondrial functions and induces aging (Figure 7). The blockade by losartan in Wnt/β‐catenin‐induced mitochondrial dysfunction and tubular cell senescence demonstrates the requirement of angiotensin system in these effects (Figures 7 and 8). Consistent with these results, it was previously reported that angiotensin II blockade could slow aging by protecting mitochondria (de Cavanagh et al., 2011). However, considering the upregulation of renin induced by RAS blockade and the sustained RAS activation in kidneys when the systemic RAS is fully blocked (Navar, Lewis, Hymel, Braam, & Mitchell, 1994), inhibition of Wnt/β‐catenin could provide a therapeutic advantage of stronger protection in aging kidneys because β‐catenin is a master regulator of RAS (Zhou, Li, et al., 2015; Zhou, Mo, et al., 2015). It is noteworthy that in an established age‐associated kidney fibrosis model, the administration of DKK1, a specific Wnt inhibitor, and the mitochondrial‐targeted antioxidant mitoQ also protect mitochondrial functions, reduce cellular senescence, and retard renal fibrosis (Figure 6). These data demonstrate the potent therapeutic effects on age‐related renal fibrosis by inhibition of Wnt/β‐catenin even after mitochondrial dysfunction and cellular senescence have occurred. Consistently, in a previous study in our group, knockdown of Wnt9a in the middle stage of disease ameliorates cellular senescence and renal fibrosis after kidney ischemia‐reperfusion injury (Luo et al., 2018).

We observed that Klotho strongly protects against d‐gal‐induced renal fibrosis and mitochondrial dysfunction. Klotho was reported as an antagonist of endogenous Wnt/β‐catenin signaling through binding to multiple Wnt proteins extracellularly in our previous study (Zhou et al., 2013). Furthermore, Klotho is predominantly localized in kidneys, which implies that among the potential therapeutic applications of Klotho, kidney diseases should be a particular focus. However, the efficacy of Klotho on mitochondrial protection in aging kidneys needs to be elucidated. Here, we observed that Klotho significantly inhibits the activation of β‐catenin and RAS activity, protects mitochondrial functions, and mitigates renal fibrosis (Figures 4 and 5). We cannot exclude that other effects of Klotho such as anti‐inflammatory and anti‐oxidative effects could also play therapeutic roles in attenuating age‐related renal fibrosis (Sopjani, Rinnerthaler, Kruja, & Dermaku‐Sopjani, 2015). To further clarify the role of Wnt/β‐catenin signaling, we adopted DKK1 and ICG‐001, which are more specific Wnt inhibitors, to treat d‐gal‐injected mice and tubular cells in culture. DKK1 inhibits mitochondrial dysfunction, cellular senescence, and renal fibrosis even in an established disease state (Figure 6), and ICG‐001 protects mitochondrial biogenesis and cell proliferation ability (Figure 8), suggesting that inhibition of Wnt/β‐catenin could be of great value in protection against age‐related mitochondrial dysfunction and renal fibrosis.

Herein, we observed that overexpression of Wnt1 or β‐catenin inhibits the mitochondrial biogenesis regulator PGC‐1α in renal tubular cells (Figure 7) and decreased mitochondrial membrane potential, oxygen consumption, and ATP production (Figure 7). Furthermore, resveratrol, an activator of PGC‐1α, and mitoQ, a mitochondrial ROS scavenger, significantly inhibit Wnt1‐induced mitochondrial mass loss and tubular cell senescence (Figure 7). These data demonstrate, in renal tubular cells, Wnt/β‐catenin signaling has a detrimental role in mitochondrial homeostasis. Our data on Wnt1 stand in contrast to previous reports that Wnt3a signaling promotes mitochondrial biogenesis (An et al., 2010; Yoon et al., 2010). The reason behind this difference may be due to difference in the cellular systems used and it does not exclude that some Wnt proteins may have opposite effects independent of β‐catenin. Consistent with this notion, we found that the interference of Wnt10b could inhibit d‐gal‐induced activation of β‐catenin and loss of PGC‐1α in tubular cells (data not shown). Taken together, these data undoubtedly show that inhibition of Wnt/β‐catenin/RAS could be an effective strategy to delay age‐related renal fibrosis. Supporting our findings, it was previously reported that Wnt/β‐catenin could activate aging in arterial, skin, and bone (Liu et al., 2007; Marchand et al., 2011).

In summary, we have shown that aberrantly expressed Wnt/β‐catenin/RAS signaling is critically involved in age‐related mitochondrial dysfunction and renal fibrotic lesions. Inhibition of Wnt/β‐catenin could provide better RAS blockade and protect mitochondrial functions, and thereby slow age‐related renal fibrosis. Although more studies are needed, these results provide proof of principle that targeted inhibition of Wnt/β‐catenin protects against aging by preventing mitochondrial dysfunction.

## 4 EXPERIMENTAL PROCEDURES

### 4.1 Animal models

Male C57BL/6 mice at 8 weeks of age and aged mice (7‐, 12‐, and 24‐month‐old mice) were obtained from the Southern Medical University Animal Center (Guangzhou, China) and housed in a standard environment with a regular light/dark cycle and free access to water and chow diet. For the accelerated aging model, male C57BL/6 mice at 8 weeks of age were administered subcutaneous injections of d‐gal (G0750; Sigma‐Aldrich) at 150 mg kg^−1^ day^−1^. In vivo expression of secreted Klotho in mice was carried out each week by a hydrodynamic‐based gene delivery approach, as described previously (Zhou, Li, et al., 2015; Zhou et al., 2013; Zhou, Mo, et al., 2015). Briefly, groups of mice were administered human secreted Klotho expression plasmid (pV5‐sKlotho; Addgene) or empty vector (pcDNA3) at 1 mg/kg by rapid injection of a large volume of DNA solution (1.6 ml) through the tail vein into the circulation within 5–10 s. Mice were euthanized at 9 weeks. For the established aging model, unilateral nephrectomy was carried out in male C57BL/6 mice at 8 weeks of age. One week after surgery, the mice were administered subcutaneous injections of d‐gal at 150 mg kg^−1^ day^−1^ for 4 weeks. After 2 and 3 weeks injection of d‐gal, a human DKK1 expression plasmid (pFlag‐DKK1; provided by Dr. Xi He, Harvard Medical School, Boston, MA) was injected at 1 mg/kg through the tail vein by a hydrodynamic‐based gene delivery approach (He et al., 2010). The mitochondria‐targeted antioxidant mitoQ (10‐1363; Focus Biomolecules) (1 mg/kg) was injected intraperitoneally every 2 days for 2 weeks after 2 weeks injection of d‐gal. Urine and kidney tissue were collected for various analyses. Detailed experimental designs are presented in Figures 4a and 6a. All animal experiments were approved by the Animal Ethics Committee at the Nanfang Hospital, Southern Medical University.

### 4.2 Cell culture and treatment

Human proximal tubular epithelial cells (HKC‐8) were provided by Dr. L. Racusen (Johns Hopkins University, Baltimore, MD, USA). Cell culture was carried out according to procedures described previously (Zhou et al., 2017). HKC‐8 cells were treated with d‐gal, human recombinant Klotho (5334‐KL; R & D Systems), losartan (61188; Sigma‐Aldrich), resveratrol (R5010; Sigma‐Aldrich), mitoQ (10‐1363; Focus Biomolecules), or ICG‐001 (847591‐62‐2; ChemLeader) at the indicated concentration. Some cells were treated with Ang II (05–23–0101; Merck Millipore) or transfected with human Wnt1 expression plasmid (pHA‐Wnt1; Upstate Biotechnology), human Wnt10b expression plasmid (pFlag‐Wnt10b), or Flag‐tagged human N‐terminal truncated, stabilized β‐catenin expression vector (pDel‐β‐catenin) constructed in our laboratory (Zhou et al., 2013). Whole‐cell lysates were prepared and subjected to real‐time PCR or Western blot analyses. Some cells were also tested by immunofluorescence or electron microscopy analyses.

Primary mouse tubular cells were isolated and cultivated as previously described (Luo et al., 2018). Briefly, the kidneys were digested in collagenase for 40 min at 37°C followed by sieving the mashed tissue in PBS. The tubular tissues were isolated using 31% Percoll gradients, resuspended, and washed twice with PBS. Finally, tubules were suspended in DMEM supplemented with 10% bovine calf serum, 50 U/ml penicillin, 50 mg/ml streptomycin, and hormone mix (5 mg/ml insulin, 1.25 ng/ml prostaglandin E1, 34 pg/ml triiodothyronine, 5 mg/ml transferrin, 1.73 ng/ml sodium selenite, 18 ng/ml hydrocortisone, and 25 ng/ml epidermal growth factor). All reagents were from Sigma‐Aldrich. Cells were grown in dishes for 4–8 days until they reached 60%–80% confluence. Media were changed on days 2 and 5, and then every 3 days thereafter.

### 4.3 Western blot analysis

Protein expression was analyzed by Western blot analysis as described previously (Zhou et al., 2017). Briefly, the kidney tissue or cultured tubular cells were homogenized in lysis buffer and their protein concentrations were measured using a Bradford assay. The homogenates were then subjected to SDS‐PAGE electrophoresis. After electrophoresis, the proteins were transferred to a PVDF (polyvinylidene fluoride) membrane (Merck Millipore), blocked in blocking buffer (1% bovine serum albumin) for 1 hr, and then incubated with primary antibodies overnight at 4°C and a secondary horseradish peroxidase‐conjugated antibody for 1 hr at room temperature. The antigen–antibody complexes were visualized using an ECL kit (Applygen). The primary antibodies used were as follows: anti‐Klotho (AF1819; R&D Systems), anti‐Kim1 (BA3537; Boster), anti‐α‐SMA (ab5694; Abcam), anti‐fibronectin (F3648; Sigma‐Aldrich), anti‐GAPDH (RM2001; Ray Antibody Biotech), anti‐Wnt1 (ab10740; Abcam), anti‐Wnt10b (ab70816; Abcam), anti‐β‐catenin (#610154; BD Transduction Laboratories), anti‐Snail1 (ab180714; Abcam), anti‐PAI‐1 (AF3828; R&D Systems), anti‐renin (sc‐27320; Santa Cruz Biotechnology), anti‐AT1 (AB15552; Merck Millipore), anti‐AGT(SAB2100072; Sigma‐Aldrich), anti‐phospo‐PGC‐1α (p‐PGC‐1α) (AF6650; R&D Systems), anti‐Cytb (SAB1304939; Sigma‐Aldrich), anti‐COX1 (SAB1301619; Sigma‐Aldrich), anti‐TFAM (GTX112760; Genetex), anti‐active β‐catenin (19807s; Cell Signaling Technology), anti‐p16^INK4A^ (ab189034; Abcam), anti‐p19^ARF^ (ab202225; Abcam), anti‐γH2AX(ab26350; Abcam), anti‐TOMM20 (ab186735; Abcam), anti‐DKK1 (BM4554; Boster), and anti‐α‐tubulin (RM2007; Ray Antibody Biotech).

### 4.4 Histology and immunohistochemical staining

Paraffin‐embedded mouse kidney sections (4 µm thickness) were prepared by a routine procedure (Luo et al., 2018). Masson trichrome staining (BA‐4079A; BASO) and Sirius red staining (DC0040; Leagene Biotechnology) were performed according to the manufacturer's protocol. Immunohistochemical staining was performed as described previously (Zhou et al., 2017). After incubation with specific antibodies, kidney sections were stained using Vector M.O.M. Immunodetection Kit according to the protocol specified by the manufacturer (Vector Laboratories). For a negative control, the primary antibody was omitted, and no staining occurred. Antibodies used were as follows: Klotho (AF1819; R&D Systems), Wnt1 (ab15251; Abcam), AT1 (AB15552; Merck Millipore), ACE (ab75762; Abcam), AGT (SAB2100072; Sigma‐Aldrich), p16^INK4A^ (ab189034; Abcam), γ‐H2AX (ab2893; Abcam), PGC‐1α (ab54481; Abcam), and DKK1 (BM4554; Boster).

### 4.5 Transmission electron microscopy

To assess mitochondrial morphology, kidney cortex and HKC‐8 cells were collected and fixed in 1.25% glutaraldehyde/0.1 M phosphate buffer. Ultrathin sections (60 nm) were prepared by a routine procedure (Xiao et al., 2017) and examined under an electron microscope (JEOL JEM‐1010).

### 4.6 SA‐β‐gal, mitoSOX, and MitoTracker staining

Frozen sections (3 μm) were used for detection of senescence via β‐galactosidase activity (#9860; Cell Signaling Technology), and mitochondria via MitoTracker deep red (M22426; Thermo Fisher) or mitochondrial ROS production via mitoSOX (M36008; Thermo Fisher) staining according to the manufacturer's instructions. Cultured cells were stained for β‐galactosidase activity and MitoTracker deep red according to the manufacturer's instructions.

### 4.7 Flow cytometry analysis

HKC‐8 cells were seeded on 6‐well plates and were given the specified treatments before staining. Then, cells were trypsinized, centrifuged at 600 g for 5 min, and then washed once with PBS. For JC‐1 staining and NAO staining, the cell pellets were suspended in 400 μl of JC‐1 (5 μM, T3168; Thermo Fisher) or NAO (5 μM, A1372; Thermo Fisher) solution, and were incubated at 37°C in the dark for 30 min. Then, cells were centrifuged, washed once, and resuspended in 400 μl of PBS. The cells were analyzed in a flow cytometry analyzer (BD FACSCalibur System). The relative mitochondrial membrane potential was calculated using the ratio of J‐aggregate/monomer (590/520 nm). Mitochondrial mass was measured by mean fluorescence intensity of NAO. For analysis of β‐gal‐positive cells, HKC‐8 cells were treated under the specified conditions, and then assessed using the FACS LacZ beta‐Galactosidase Intracellular Detection Kit (ab189816; Abcam) according to the manufacturer's protocol. Propidium iodide (PI) was used to exclude dead cells.

### 4.8 Measurements of mitochondrial oxygen consumption rate

The rates of mitochondrial oxygen consumption in HKC‐8 cells were measured with a Seahorse XFe96 Extracellular Flux Analyzer (Seahorse Bioscience), using the XF Cell Mito Stress Test Kit (#103015; Seahorse Bioscience). Approximately 3 × 10^4^ cells were seeded per well in a Seahorse 96‐well XF Cell Culture Microplate. XF assays were performed according to the manufacturer's instructions with the addition of oligomycin (1 μM), FCCP (0.5 μM) and antimycin A and rotenone (0.5 μM each). Parameters of basal OCR, maximal OCR, ATP‐linked OCR, and the reserve capacity were analyzed according to the manufacturer's protocol.

### 4.9 BrdU incorporation assay

HKC‐8 cells were cultured and stimulated as indicated. BrdU (B5002; Sigma‐Aldrich) (10 μM) was added 12 hr before collection. Cells were fixed with 70% ice–methanol at 4°C for 20 min, and the DNA was denatured with 2.5 N aqueous HCl for 20 min. The mixture was neutralized with 0.1 M boric acid for 10 min and then incubated with 3% H_2_O_2_/PBS for 20 min. After blocking with 10% donkey serum for 60 min, the slides were immunostained with a primary antibody against BrdU (MAB4072; Sigma‐Aldrich) overnight at 4°C and then stained with a Cy3‐conjugated secondary antibody (Jackson ImmunoResearch Laboratories). Images were taken by fluorescence microscopy (Leica DMi8; Leica Microsystems).

### 4.10 RT and real‐time PCR

Total RNA isolation was carried out using the TRIzol RNA isolation system (Life Technologies) according to the manufacturer's instructions. The first strand of complementary DNA was synthesized using 1 μg of RNA in 20 μl of reaction buffer containing AMV‐RT (Life Technologies) and random primers at 42°C for 60 min. Real‐time PCR was performed using a Platinum SYBR Green qPCR SuperMix‐UDG kit (Invitrogen). The sequences of the primer pairs are shown in Table S1. mtDNA analysis was performed as previously reported (Gomes et al., 2013). Briefly, total DNA was extracted with a DNeasy blood and tissue kit (Qiagen) according to the manufacturer's instructions. mtDNA was amplified using primers for the mitochondrial cytochrome *c* oxidase subunit 2 (COX2) gene and normalized to ribosomal protein s18 (RSP18). Primer pairs for both mouse and human Wnt genes were described previously (He et al., 2009; Zhou et al., 2013).

### 4.11 Immunofluorescence staining

Kidney cryosections (3 µm) were fixed with 4% paraformaldehyde for 15 min at room temperature. HKC‐8 cells cultured on coverslips were fixed with cold methanol:acetone (1:1) for 10 min at room temperature. After blocking with 10% donkey serum for 60 min, the slides were immunostained with primary antibodies against LTL (FL‐1321; Vectorlab), NCC (ab3553; Abcam), AQP3 (ab125219; Abcam), mannose R (ab64693; Abcam), PDGFRβ (Sc‐432; Santa Cruz Biotechnology), EMCN (Endomucin) (AF4666; R&D System), E‐cadherin (ab76055; Abcam), Klotho (R&D System), β‐catenin (Abcam), collagen I (Boster), and fibronectin (Sigma‐Aldrich), as aforementioned. The slides were then stained with a Cy3‐ or Cy2‐conjugated secondary antibody (Jackson ImmunoResearch Laboratories), and mounted with Vectashield antifade mounting media (Vectorlab). Negative controls were performed by incubation with secondary antibodies alone (omitting primary antibody). Nuclei were stained with DAPI (Sigma‐Aldrich) according to manufacturer's instructions. Images were captured using fluorescence microscopy (Leica DMi8; Leica Microsystems) or a Leica TCS‐SP8 confocal microscope.

### 4.12 Statistical analyses

All data examined were expressed as mean ± *SEM*. Statistical analysis of the data was carried out using SPSS 13.0 (SPSS Inc.). Comparison between groups was made using one‐way ANOVA followed by Student–Newman–Keuls test or Dunnett's T3 procedure. *p* < .05 was considered significant.

## CONFLICT OF INTERESTS

None declared.

## AUTHORS’ CONTRIBUTIONS

Jinhua Miao, Jiafeng Liu, Jing Niu, Yunfang Zhang, Congwei Luo, Yahong Liu, Chuanjing Li, and Hongyan Li conducted the experiments and prepared the materials involved in this study. Lili Zhou conceived this study. Fan Fan Hou, Youhua Liu, and Lili Zhou participated in its design and coordination. Lili Zhou, Jinhua Miao, Jiafeng Liu, Jing Niu, and Yunfang Zhang contributed to the analysis and interpretation of the data. Lili Zhou drafted the manuscript. Jinhua Miao, Jiafeng Liu, Jing Niu, Yunfang Zhang, and Peiliang Yang helped to revise the manuscript. All authors read and approved the final manuscript.
