# Corrigendum to: Wnt/β‐catenin/RAS signaling mediates age‐related renal fibrosis and is associated with mitochondrial dysfunction

**DOI:** 10.1111/acel.13816

**Published:** 2023-04-08

Miao, J. , Liu, J. , Niu, J. , Zhang, Y. , Shen, W. , Luo, C. , Liu, Y. , Li, C. , Li, H. , Yang, P. , Liu, Y. , Hou, F.F. , Zhou, L. Wnt/β‐catenin/RAS signaling mediates age‐related renal fibrosis and is associated with mitochondrial dysfunction. Aging Cell. 2019;18(5):e13004. 10.1111/acel.13004.31318148PMC6718575

In the above published article, the authors mistakenly placed a duplicated image of PGC‐1α staining for the CTL and D‐gal/Klotho groups in Figure 5b. The corrected version of Figure 5b now contains the correct PGC‐1α staining images, and it is shown below.

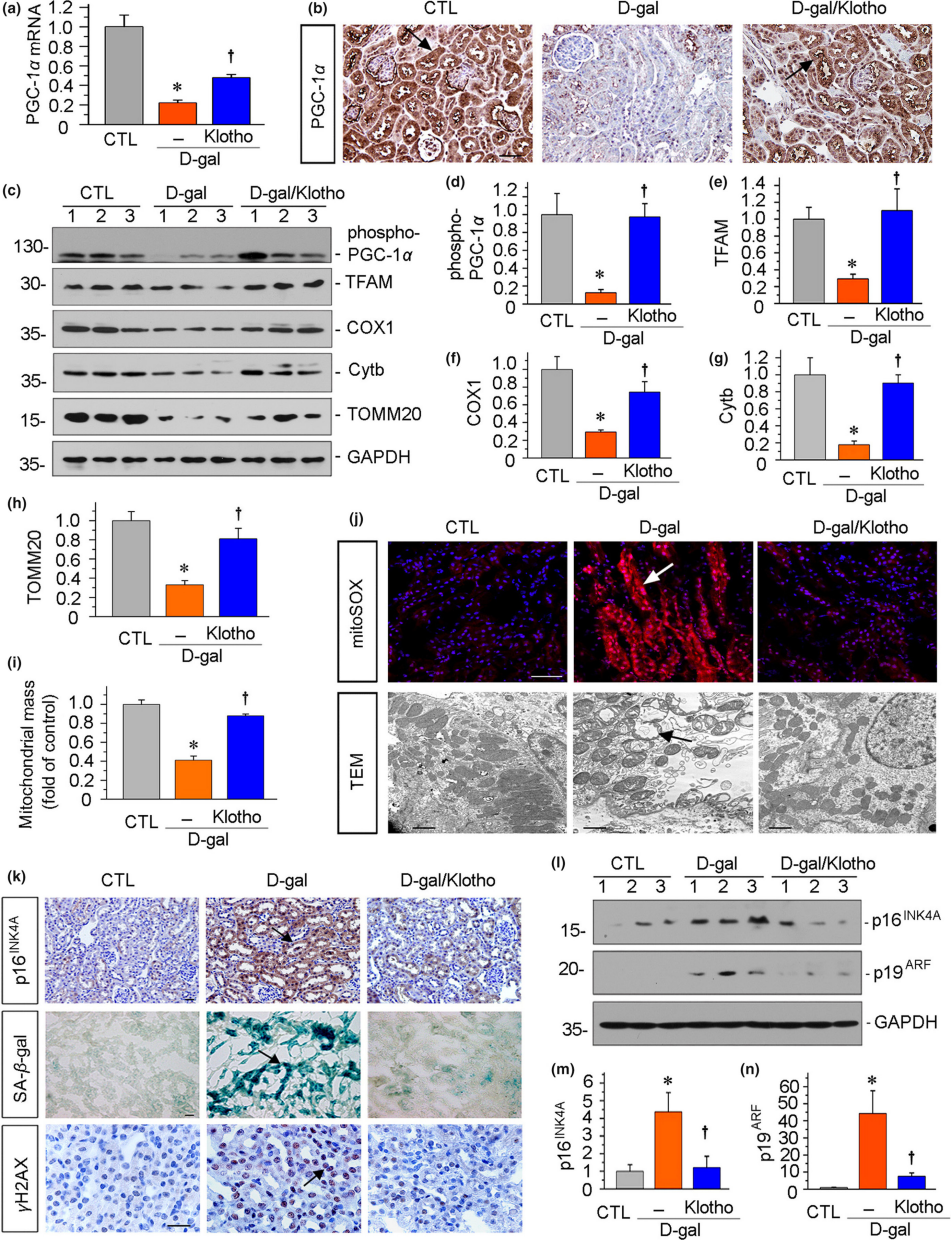

The authors would like to apologize for the inconvenience caused.

