# Supplementary figures and images for: Wnt/β‐catenin/RAS signaling mediates age‐related renal fibrosis and is associated with mitochondrial dysfunction
Source: Aging Cell. 2019 Jul 18;18(5):e13004. doi: 10.1111/acel.13004 (PMC6718575; doi:10.1111/acel.13004)

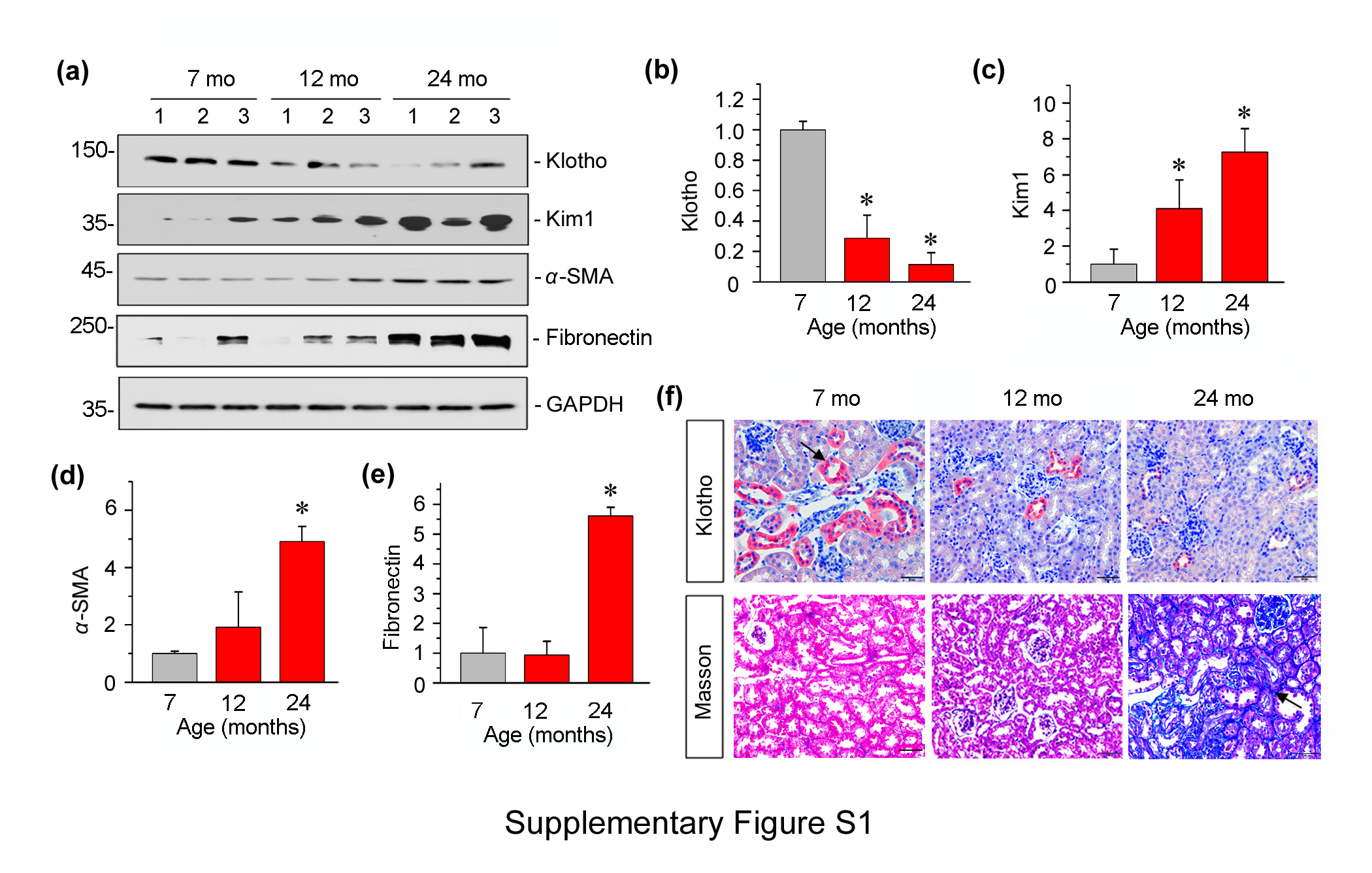

Supplement: Supplementary file 1 [file ACEL-18-e13004-s001.jpg]

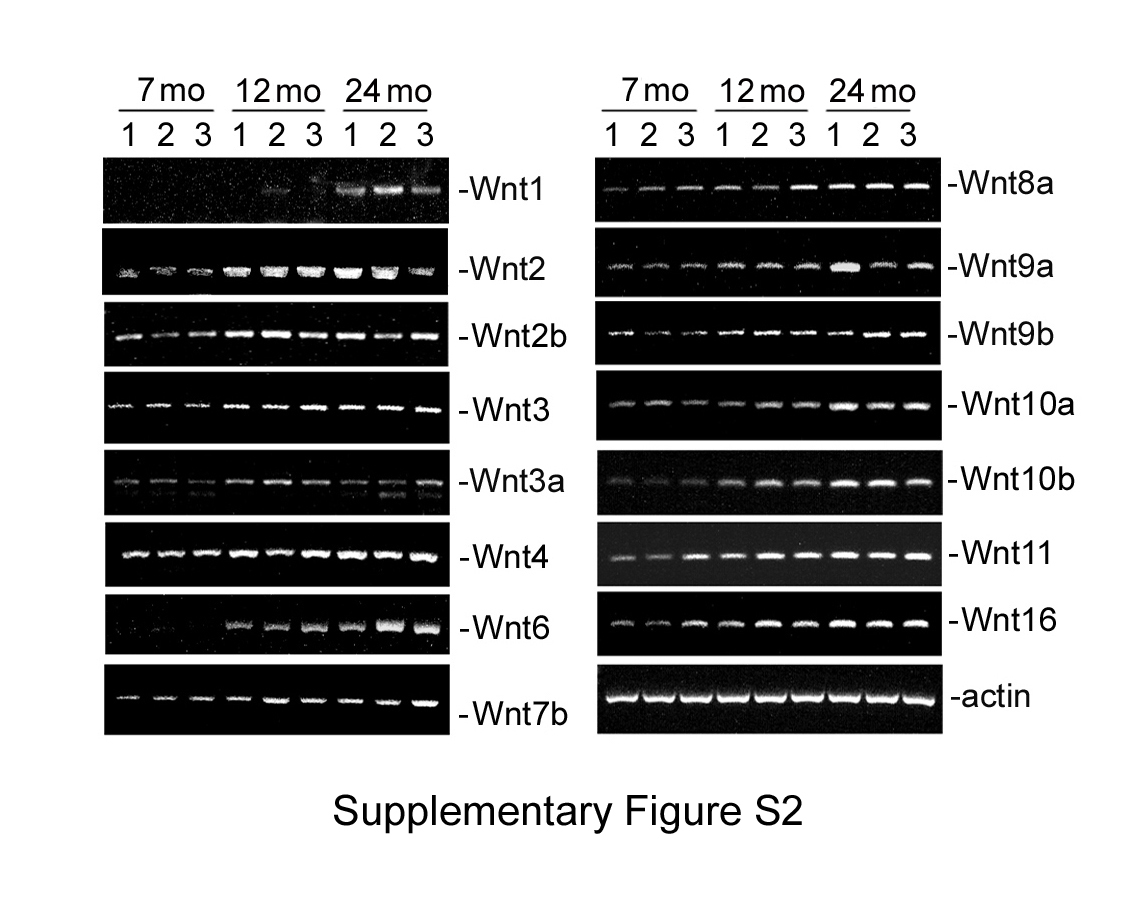

Supplement: Supplementary file 2 [file ACEL-18-e13004-s002.jpg]

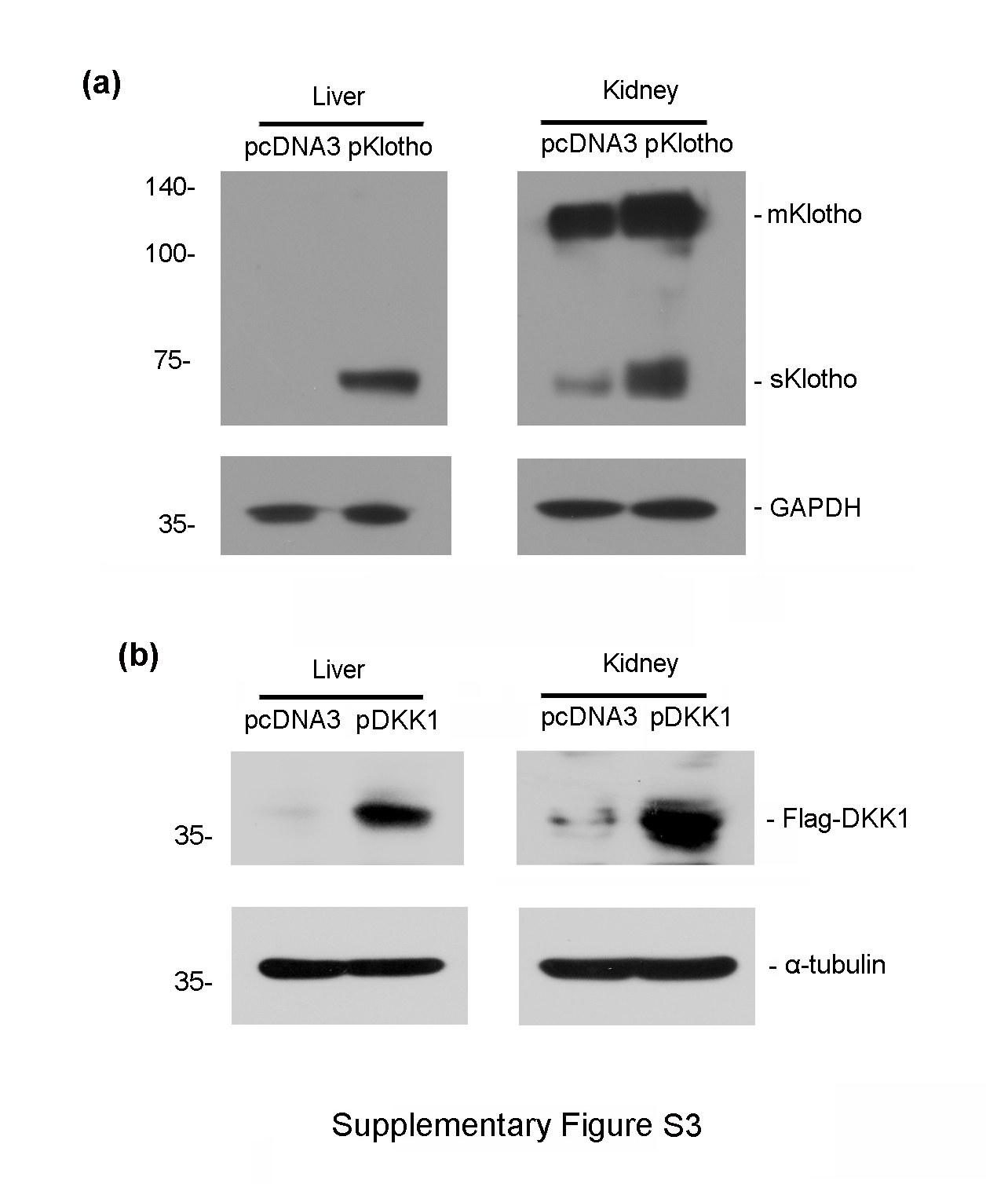

Supplement: Supplementary file 3 [file ACEL-18-e13004-s003.jpg]

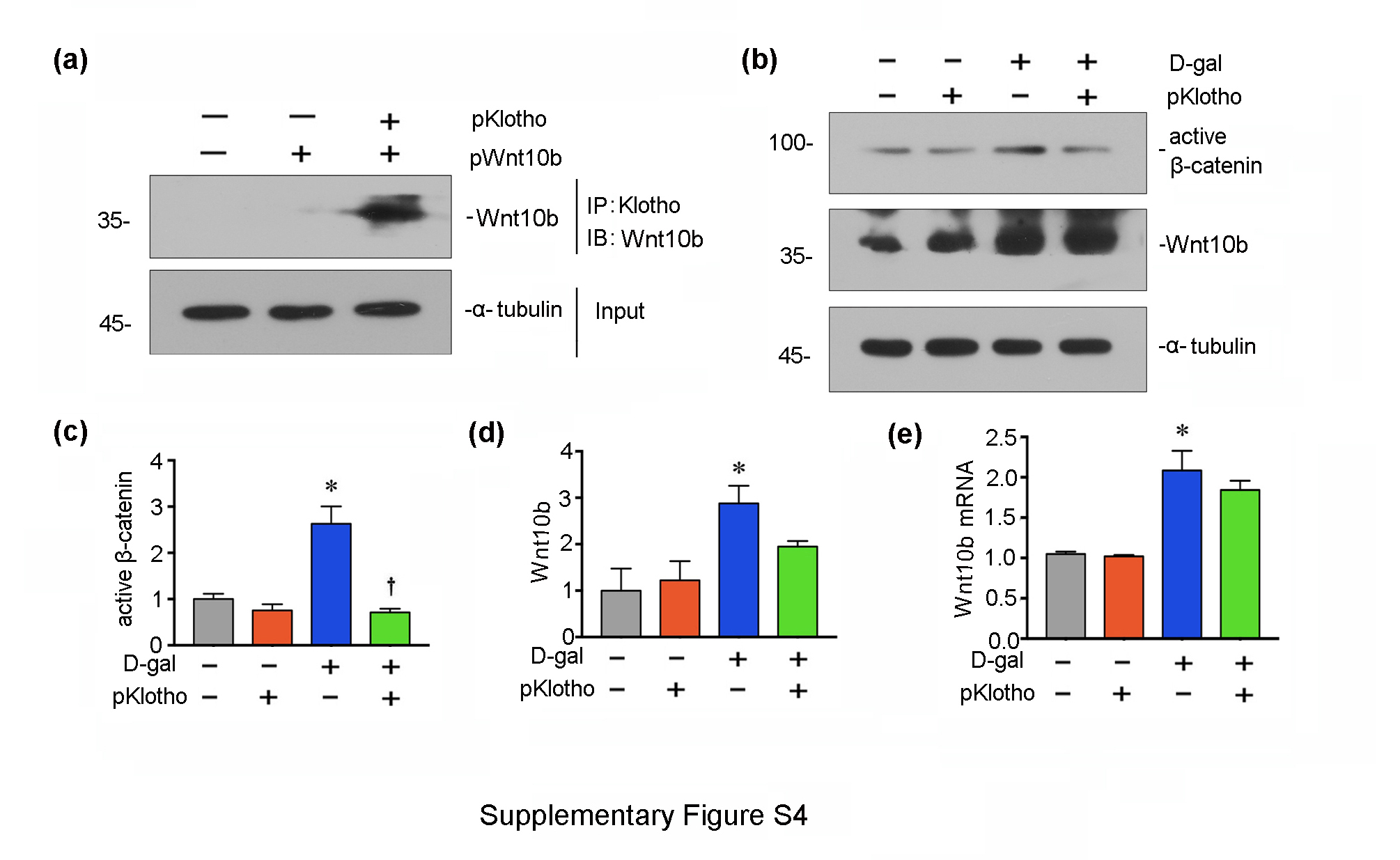

Supplement: Supplementary file 4 [file ACEL-18-e13004-s004.jpg]

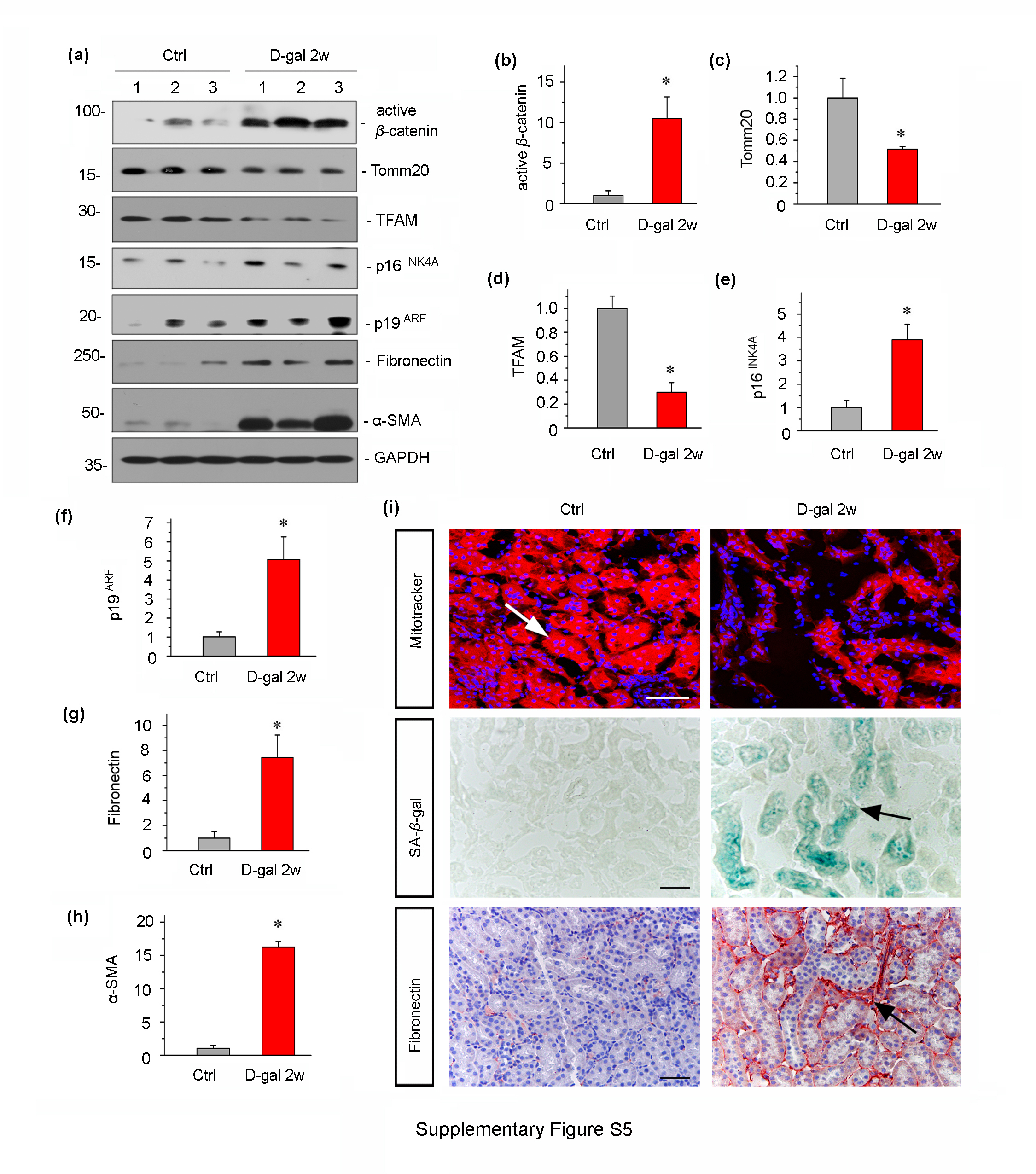

Supplement: Supplementary file 5 [file ACEL-18-e13004-s005.jpg]

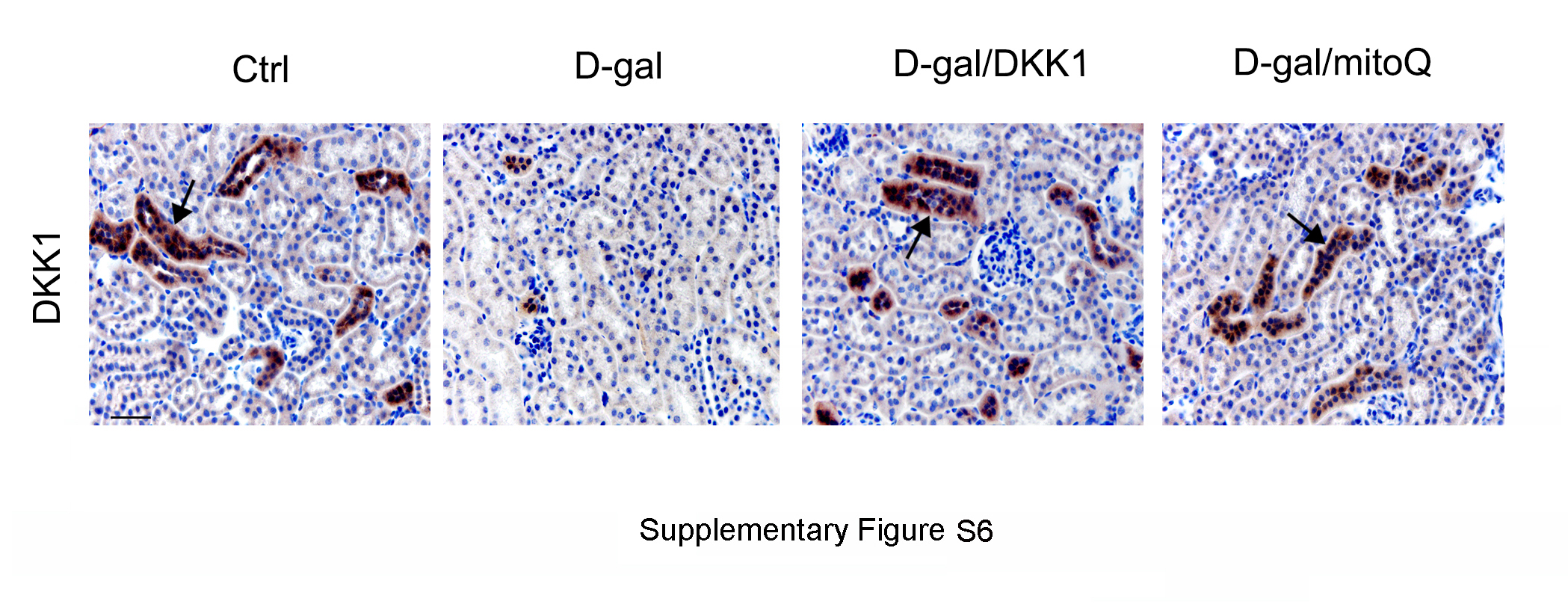

Supplement: Supplementary file 6 [file ACEL-18-e13004-s006.jpg]

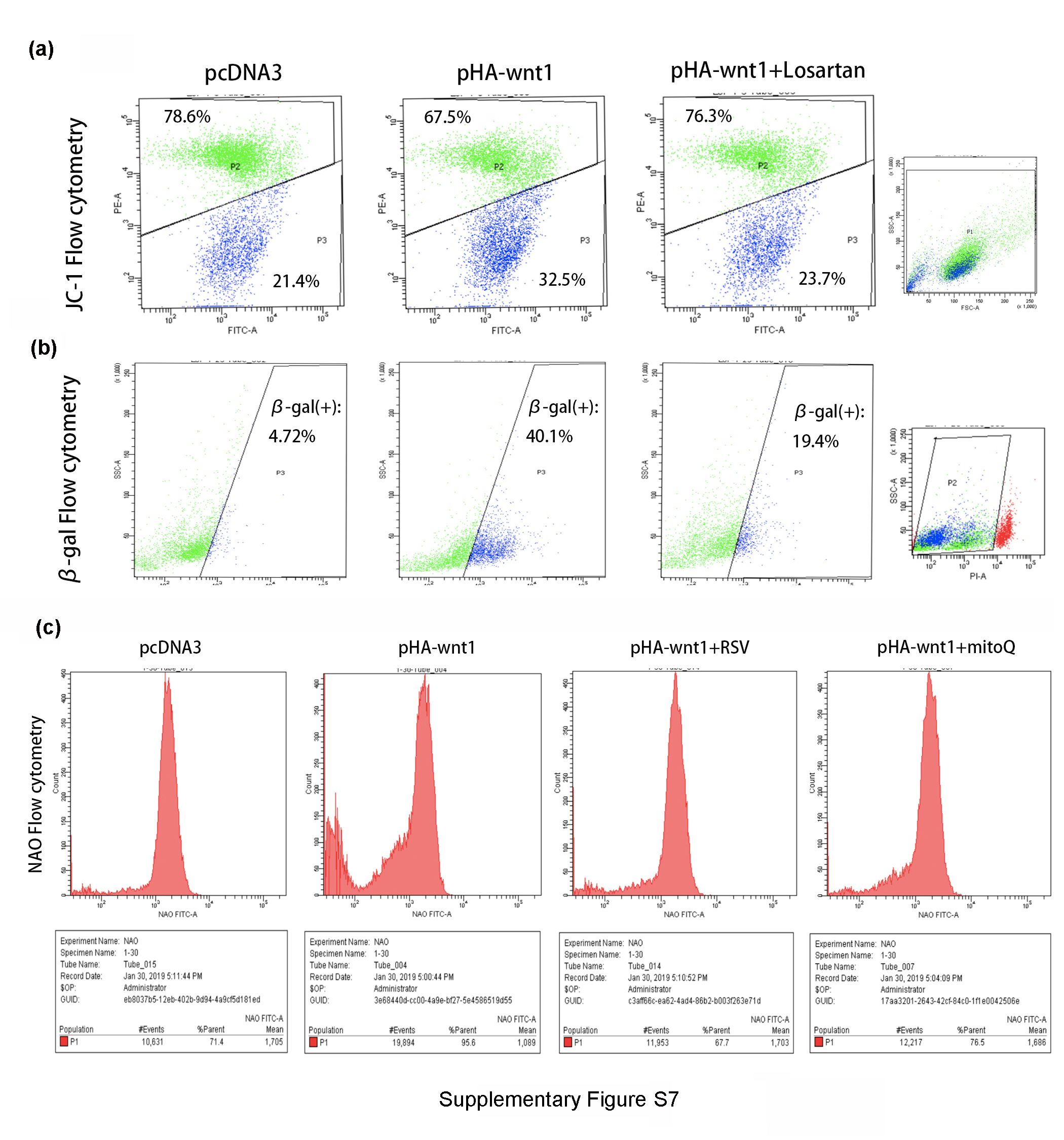

Supplement: Supplementary file 7 [file ACEL-18-e13004-s007.jpg]

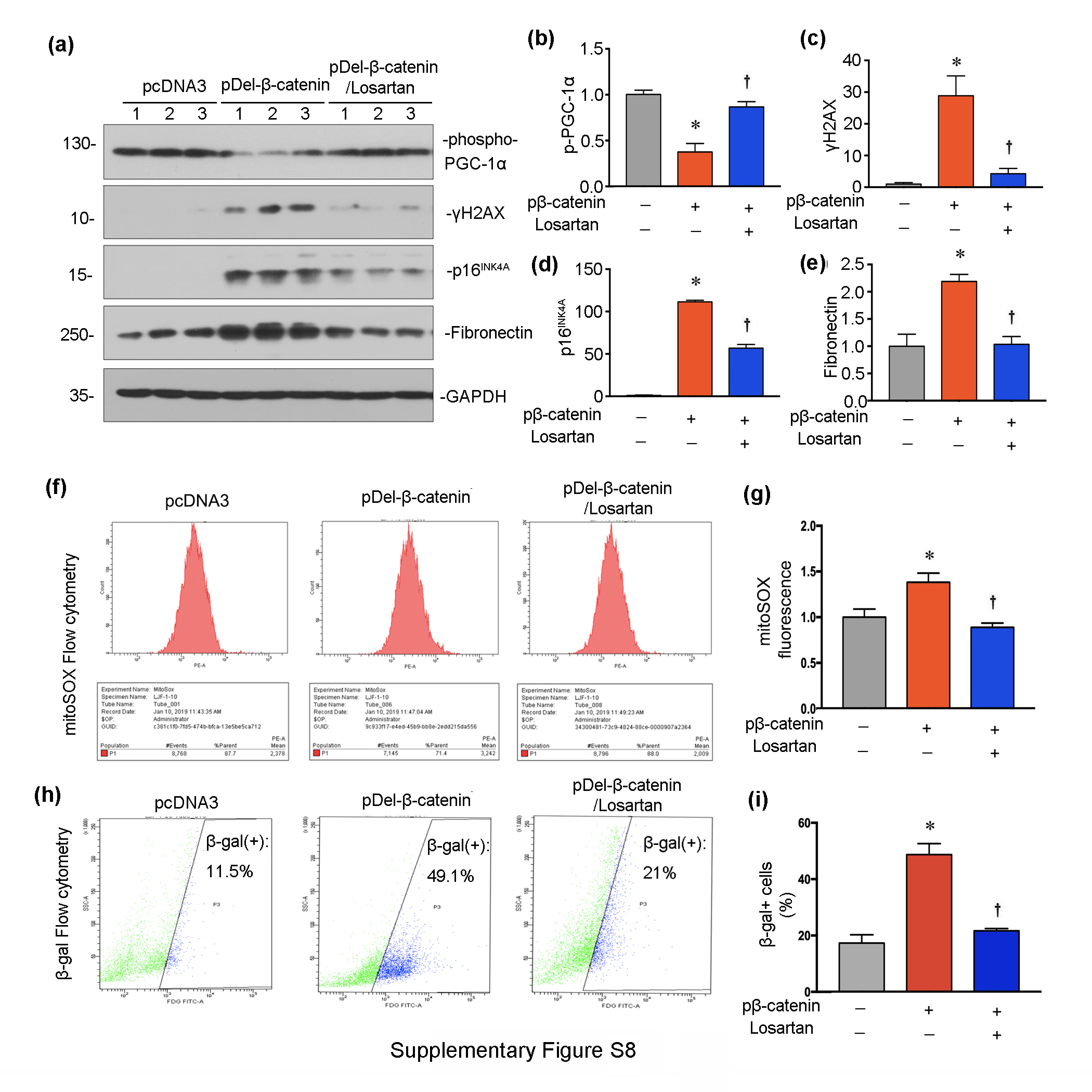

Supplement: Supplementary file 8 [file ACEL-18-e13004-s008.jpg]
