# Supplementary material for: Wnt/β‐catenin/RAS signaling mediates age‐related renal fibrosis and is associated with mitochondrial dysfunction
Source: Aging Cell. 2019 Jul 18;18(5):e13004. doi: 10.1111/acel.13004 (PMC6718575; doi:10.1111/acel.13004)
Supplement: Supplementary file 9 [file ACEL-18-e13004-s009.docx]

**Supporting Information Table 1**

Nucleotide sequences of the primers used for PCR

| **Gene** | **Primer Sequence 5’ to 3’** | |
| --- | --- | --- |
|  | **Forward** | **Reverse** |
| MMP-7-Mouse | TAGGCGGAGATGCTCACTTT | TTCTGAATGCCTGCAATGTC |
| TFAM-Human | CGCTCCCCCTTCAGTTTTGT | CCAACGCTGGGCAATTCTTC |
| Cytb-Human | CAATTCTCCGATCCGTCCCT | GCTTACTGGTTGTCCTCCGA |
| COX1-Human | AAAACCCCCTGCCATAACCC | GTGTTGAGGTTGCGGTCTGT |
| COX2-Human | AGACGAGGTCAACGATCCCT | TGTCAACGTCAAGGAGTCGC |
| PGC-1α-Mouse | CACCAAACCCACAGAAAACAG | GGGTCAGAGGAAGAGATAAAGTTG |
| TFAM-Mouse | CACCCAGATGCAAAACTTTCAG | CTGCTCTTTATACTTGCTCACAG |
| Cytb-Mouse | CCCACCCCATATTAAACCCG | GAGGTATGAAGGAAAGGTATTAGGG |
| ATP6-Mouse | TCCCAATCGTTGTAGCCATC | TGTTGGAAAGAATGGAGACGG |
| COX1-Mouse | CCCAGATATAGCATTCCCACG | ACTGTTCATCCTGTTCCTGC |
| COX2-Mouse | AGTTGATAACCGAGTCGTTCTG | CTGTTGCTTGATTTAGTCGGC |
| mtDNA(COX2) | ATAACCGAGTCGTTCTGCCAAT | TTTCAGAGCATTGGCCATAGAA |
| mtDNA(RSP18) | TGTGTTAGGGGACTGGTGGACA | CATCACCCACTTACCCCCAAAA |
| β-actin-Mouse | cagctgagagggaaatcgtg | cgttgccaatagtgatgacc |
| β-actin-Human | CTCACCATGGATGATGATATCGC | AGGAATCCTTCTGACCCATGC |
